# A generalized approach for estimating the Pace of Aging

**DOI:** 10.1093/gerona/glag205

**Published:** 2026-08-20

**Authors:** Marije H Sluiskes, Joris Deelen, Hein Putter, Sara Hägg, Mar Rodríguez-Girondo

**Affiliations:** Biomedical Data Sciences, Leiden University Medical Center, Leiden, The Netherlands; Medical Epidemiology and Biostatistics, Karolinska Institutet, Stockholm, Sweden; Biomedical Data Sciences, Leiden University Medical Center, Leiden, The Netherlands; Biomedical Data Sciences, Leiden University Medical Center, Leiden, The Netherlands; Medical Epidemiology and Biostatistics, Karolinska Institutet, Stockholm, Sweden; Biomedical Data Sciences, Leiden University Medical Center, Leiden, The Netherlands; (Medical Sciences Section)

**Keywords:** Biomarkers of aging, Age acceleration, Longitudinal data, Mixed-effects models, Biological age

## Abstract

**Background:**

Quantifying the rate of aging is essential for understanding age-related physiological decline and predicting late-life outcomes. The Pace of Aging (PoA) framework addresses this by modeling longitudinal changes across multiple biomarkers. However, the original Dunedin PoA assigns equal weight to all biomarkers and was derived from a young cohort with limited mortality follow-up, which restricts its applicability in older populations and in settings where biomarker relevance is uncertain.

**Methods:**

We developed a weighted Pace of Aging (wPoA) using data from the Swedish Adoption/Twin Study of Aging (SATSA), a longitudinal cohort of older adults with up to nine waves of biomarker data collected over three decades. Using mixed-effects models, we estimated individual random intercepts and slopes for 10 biomarkers and assigned weights to these random effects based on their association with time-to-mortality. This mortality-informed weighting distinguishes the wPoA from the original Dunedin PoA.

**Results:**

We found that wPoA was positively correlated with established biological age predictors and more strongly correlated with low-dimensional predictors such as the Functional Aging Index (FAA) and the Frailty Index (FI). Modest correlations across all considered predictors suggest that each measure captures distinct aspects of biological aging.

**Conclusions:**

In conclusion, the wPoA framework introduces a data-driven weighting of random effects and is particularly well-suited for aging cohorts and in settings where marker relevance is uncertain a priori, such as high-dimensional or omics-based contexts. This work provides a foundation for future single-timepoint surrogates that can also be used in settings lacking rich longitudinal data.

## Introduction

People of the same chronological age do not age at the same rate: some remain healthy and live long lives, while others experience early functional decline, develop various diseases, and die prematurely. This observation of varying aging rates is closely linked to the idea of ‘biological age.’ Unlike chronological age, which is simply the number of years a person has lived, biological age reflects the function of the body taking into account the cumulative effects of genetics, lifestyle, and environmental factors.

Most existing biological aging predictors are based on single-timepoint measurements of candidate biomarkers of aging.^1^ They tend to be trained on chronological age or, more recently, prospective mortality. However, this approach may miss a crucial dimension of aging: its pace. Rather than estimating how old someone is biologically at one moment in time, a potentially more informative strategy is to assess how fast someone is aging. This requires repeated measurements of biomarkers over time, enabling the estimation of intra-individual changes. As such, there is growing interest in developing predictors based on repeated measurement data, which may be viewed as a third generation of biological aging clocks,^2^ after the first-generation age-trained and second-generation mortality-trained ones.

Although still scarce, datasets with repeated measurements of candidate biomarkers of biological age are becoming increasingly available. However, the number of studies using such data in aging speed prediction remains limited. The Pace of Aging (PoA)^3^ pioneered the use of repeated measures to quantify the rate of individual physiological decline over time. A few years later, the same group proposed a single timepoint methylation-based predictor of PoA, called DunedinPACE,^4^^,^^5^ which can be used by others who do not have access to rich repeated measurement data.

The PoA predictor was trained on data from the Dunedin study, a population-representative birth cohort of about one thousand young adults from Dunedin, New Zealand, using 19 biomarkers that are known risk factors or correlates of chronic disease and mortality. A faster PoA has been associated with several age-related outcomes,^6–9^ in subsequent studies. A key methodological feature of the PoA is its reliance on individual-specific slopes of standardized biomarkers, fitted with mixed-effects models and interpreted as individual-specific rates of change. The PoA is computed as their sum, such that biomarkers with larger rates of change, after standardization, contribute more, implicitly assuming that longitudinal changes across biomarkers are equally informative of the underlying aging process.

Similar approaches have been applied in other cohorts. Kuo et al. (2022) used an approach based on averaging random slopes in the Baltimore Longitudinal Study of Aging (BLSA).^10^ More recently, Balachandran et al.^11^ proposed an adaptation of PoA for older populations that allow for non-linear biomarker trajectories across age.

In this paper, we sought to develop a new PoA predictor using data from the Swedish Adoption/Twin Study of Aging (SATSA),^12^ a longitudinal study of older twins with up to ten testing waves at approximately three-year intervals. Because in SATSA, a different set of biomarkers was measured than in the Dunedin study, it was not possible to apply the original PoA directly. This provided an opportunity to revisit and extend the underlying methodology.

Our main contribution is to introduce a data-driven weighting scheme for the biomarker-specific rates of change. Specifically, we estimate random slopes from mixed-effects models and assign weights based on their association with time-to-mortality. This approach allows candidate biomarkers to be included without prior assumptions about their relevance: those that are less informative will naturally receive lower weights. As in previous work in older populations,^11^ we allow for non-linear biomarker trajectories across age.

The idea of assigning data-driven weights to the random effects underlying PoA was already discussed in the original paper,^3^ but has not been previously implemented.

The theoretical motivation and statistical implementation of the weighted Pace of Aging (wPoA) are presented in Methods. This includes a detailed discussion of the conceptual choices that were made. In Results, we examine the association of wPoA with aging-related health outcomes. Here, we also compare the correlation of wPoA with other (age-adjusted) established biological aging measures. Our overall goal is to refine the sensitivity and validity of PoA predictors derived from repeated measurement data.

## Methods

This section describes the construction of the novel wPoA. Since our wPoA is an extension of the original PoA, the construction of the original PoA is described first to better highlight similarities and differences. After wPoA is introduced, this section discusses the methodological choices and technicalities of the new predictor. It is also discussed how the performance of wPoA can be evaluated.

### Original Dunedin Pace of Aging

PoA was built using data from the Dunedin study, a population-representative birth cohort of 1,037 young adults. Two versions have been proposed: the original predictor^3^ and an updated version,^4^ created after data on a fourth measurement became available.

This most recent version of PoA is based on 19 biomarkers, which are known risk factors or correlates of chronic disease and mortality, measured at four different points in time (at ages 26, 32, 38, and 45). Barely any mortality had been observed in this cohort: attrition by death was less than three percent at age 45.

From the Dunedin dataset, PoA was calculated from these biomarkers in three steps:

1. All markers were standardized to have the same scale and direction: i.e., all markers have mean 0 and standard deviation 1, and higher values indicate a worse/older state.
2. For each of the 19 markers, a mixed-effects model was fitted. The following model was fitted for each marker *X*:
  $$X_{ti}=\beta_{0}+\beta_{1}{\mathrm{Age}}_{ti}+v_{0i}+v_{1i}{\mathrm{Age}}_{ti}+\varepsilon_{ti}$$
  where $X_{ti}\;$denotes marker *X* measured for individual *i* at time point *t*, $\beta_{0}\;$and $\beta_{1}\;$are the fixed intercepts and slopes for the cohort, and $v_{0i}\;$and $v_{1i}\;$are the random intercept and slope specific to individual *i*. The random intercepts $v_{0i}\;$and random slopes $v_{1i}\;$are assumed to be independent and normally distributed with means zero and variances $\sigma_{v_{0}}^{2}$ and $\sigma_{v_{1}}^{2}$, respectively, and are independent of the residual errors $\varepsilon_{ti}∼N(0,\sigma_{\varepsilon }^{2})$.
3. PoA was calculated by summing all 19 individual-specific slopes:
  $${\mathrm{PoA}}_{i}=\sum_{X}{{(\beta }_{1}+\hat{v}}_{1iX})$$

### Data

The new wPoA was constructed using data from the SATSA,^12^ a longitudinal study of Swedish twins, initiated to study the effects on aging from being reared apart or together before the age of 10 years. Nine occasions of in-person testing (IPT) were conducted between 1986 and 2014 at approximately three-year intervals. Each IPT consisted of an extensive data collection procedure, including physical tests and a blood draw. The study enrolled twins aged 50 or older at baseline, and the last testing occurred in 2014. Individuals were then followed in national registries for mortality until 2025. Participants were invited for each IPT, regardless of whether they had missed any previous ones. In total, 859 study participants attended at least one IPT session.

The following ten biomarkers were included in the construction of the wPoA: waist-hip ratio, grip strength, MMSE7, flexibility, balance, FEV1, FVC, the ratio FEV1/FVC, blood hemoglobin, and blood sugar. Variable selection was based on the following criteria: a median number of measurements of at least four, a monotone association with mortality throughout the lifespan, and an observed association with age that agrees with the expected direction. Values more than five standard deviations from the mean were removed. See Supplementary Information, Note 1, for spaghetti plots of each marker and detailed information on variable definitions and exclusions. Individuals with complete measurements on all ten biomarkers on at least one IPT were eligible for inclusion. This resulted in a final sample of 745 individuals.

### Construction weighted Pace of Aging

The construction of wPoA from the SATSA data is as follows:

1. For each marker *X*, the following mixed model was fitted:
  $$X_{\mathrm{tij}}=\beta_{0}+\sum_{m=1}^{3}\beta_{m}{ns}_{m}({\mathrm{Age}}_{ti})+\gamma {\mathrm{Sex}}_{i}+\sum_{m=1}^{3}\delta_{m}[{ns}_{m}({\mathrm{Age}}_{ti})\times {\mathrm{Sex}}_{i}]+u_{0j}+v_{0i}+v_{1i}{\mathrm{Age}}_{ti}+\varepsilon_{\mathrm{tij}}$$
  where $X_{\mathrm{tij}}\;$denotes marker *X* measured at time point *t* for individual *i* (of twin pair *j*, left out in subsequent subscripts for simplicity); $\beta_{0}\;$is the fixed effects intercept for the cohort, $\beta_{1},\ldots ,\;\beta_{3}$ are the fixed effects associated with the spline of age (one coefficient per spline basis), γ is the fixed effect for sex, $\delta_{1},\ldots ,\;\delta_{3}$ are the fixed effects associated with the interaction between sex and the spline of age; *u*_0__*j*_ is the random intercept specific to twin pair *j*; and *v*_0__*i*_ and *v*_1__*i*_ are the random intercept and slope specific to individual *i*.
2. The estimated subject-specific random slopes $v_{1i}$ from 10 markers were subsequently included as covariates in a survival model with time-to-mortality as the event of interest. The survival model that was fitted is an accelerated failure time (AFT) model^13^ relating covariates to the logarithm of survival time:
  $$\log\left(T_{i}\right)=\alpha_{0}+\sum_{k=1}^{10}\alpha_{1k}v_{1i}^{\left(k\right)}+\theta {\mathrm{Sex}}_{i}+\varepsilon_{i}$$
  where *T_i_* denotes the survival time to mortality for individual *i*, $v_{1i}^{\left(k\right)}\;$is the random slope for marker *k* in individual *i*, *α*_1__*k*_ is the corresponding regression coefficient, and θ is the coefficient for sex. *ε_i_* is the error term, assumed to follow a specific probability distribution (which in turn determines the assumed distribution of the event times *T*).
3. The wPoA is calculated by obtaining the linear predictor excluding the sex effect, denoted by ρ and taking the exponent:
  $${\mathrm{wPoA}}_{i}=\text{exp}\;[\rho ],\text{where}\;\rho =\sum_{k=1}^{10}\alpha_{1k}{\hat{v}}_{1i}^{\left(k\right)}.$$

**Figure 1:**
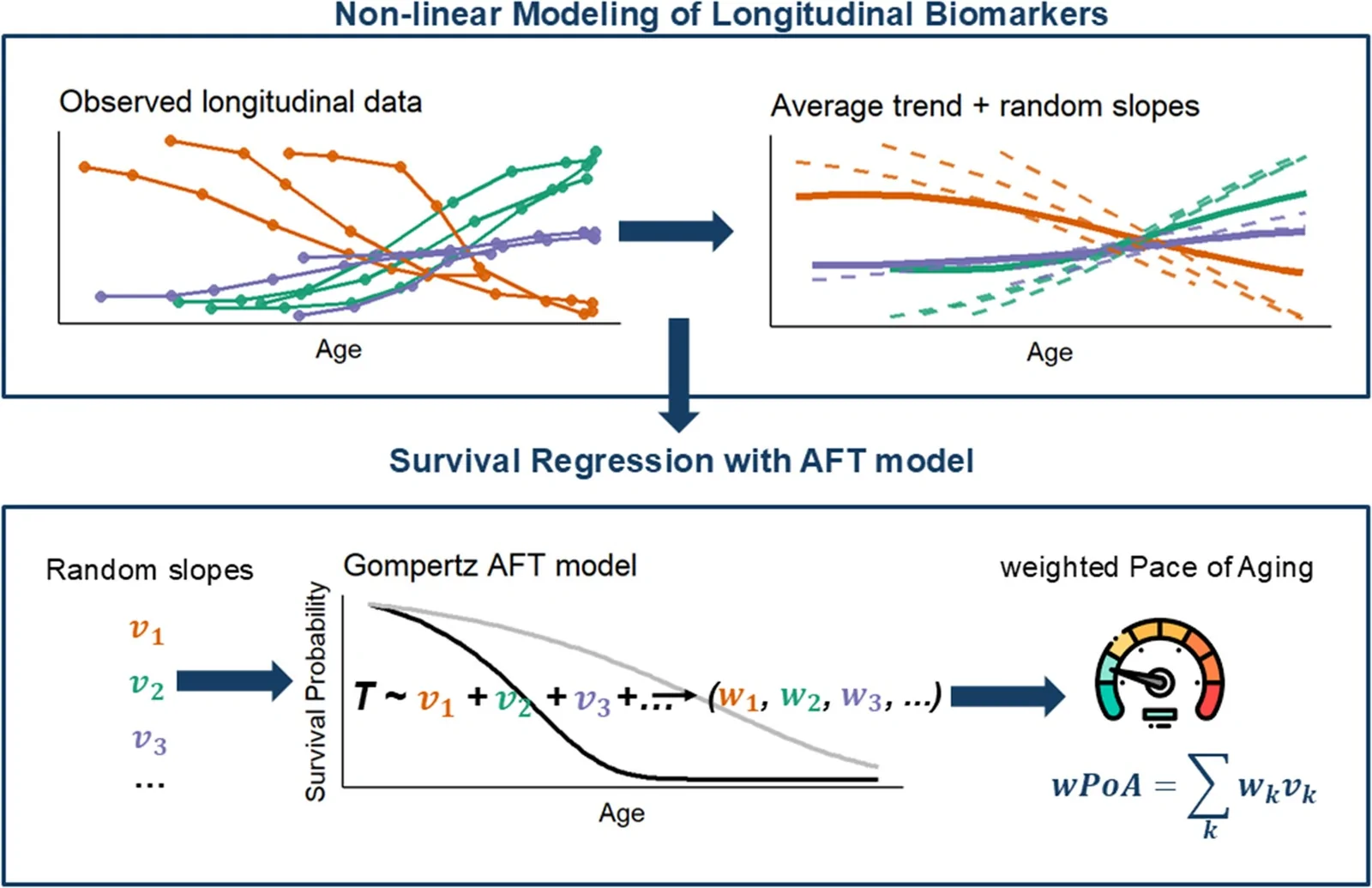
Illustration of the construction of the weighted Pace of Aging.

See Figure 1 for a visual representation of the construction of the wPoA. Plots of the fixed effects of each included marker (i.e., $\beta_{0}+\sum_{m=1}^{3}\beta_{m}{ns}_{m}({\mathrm{Age}}_{ti})+\gamma {\mathrm{Sex}}_{i}+\sum_{m=1}^{3}\delta_{m}[{ns}_{m}({\mathrm{Age}}_{ti})\times {\mathrm{Sex}}_{i}]$) can be found in Supplementary Information, Note 2. This includes the markers that were not included in step 2 due to an unexpected relation with age (cholesterol, ApoA1, ApoB, and the ratio ApoB/ApoA1), attributed to lipid-altering medication use.

### Technical overview

Compared to the original PoA construction described in section Original Dunedin Pace of Aging, the markers used in the construction of the wPoA do not have to be standardized in order to exhibit the same direction with age. Such a transformation is only necessary when random effects are simply summed: in such a case, it is essential that higher values always represent a higher risk (or vice versa). If predictors are weighted, the direction of the weights adjusts for the fact that some markers are negatively associated with age and others positively.

There are several differences in the mixed-effects model formulation between the original and wPoA. First, an additional level of random effects is modeled in the mixed models underlying the wPoA, due to the twin structure in the SATSA data. The individual-specific random intercepts are nested within the twin-specific random intercepts. In addition, for the wPoA it was decided to model age with a third-degree spline. This allows for a possibly nonlinear relation between a marker and age. Since the observed association may differ between men and women, sex and the interaction between sex and age were included in the model as well.

It is important to consider potential non-monotonicity in the relationship between individual markers and mortality, as this also implies non-monotonicity in the effects of the random markers on time-to-mortality (step 2 of the wPoA construction), while our AFT model assumes time-constant effects. This is specifically a concern in an older population such as SATSA, as it is known that in older people the effect of some markers on mortality may indeed reverse direction. We had prior knowledge that this is the case for BMI^14^ and blood triglyceride levels:^15^ from a certain age onward, higher values no longer pose a risk but may in fact protect. Hence, these markers were excluded.

Including sex as a covariate in the survival model in step 2 of the wPoA construction is essential: as women tend to live longer than men; the survival model would simply be predicting lifespan by considering gender-specific differences in random effects if sex were not included as a covariate. Instead, the wPoA should be predictive of remaining lifespan, conditional on sex.

We chose to fit a Gompertz AFT model^16^ in step 2, which is a specific type of survival analysis model. Although the AFT model is less commonly used than the Cox Proportional Hazards model, it is conceptually closer to the idea of ‘aging as a clock’: the AFT model assumes that the effect of the linear predictor ρ on the baseline survival curve is to shrink or stretch this curve by a factor exp(ρ): $S(t|\rho )=S_{0}(t\times \text{exp}(\rho ))$. Since wPoA is defined as exp(ρ), it can directly be interpreted as the aging speed: e.g., a wPoA of 1.1 means that an individual is aging at 1.1 times the speed of the average person. The choice of Gompertz distribution for the baseline survival is justified by its well-established suitability for modeling adult human mortality. See for more details on the AFT model and its relevance to aging predictors.^16^

**Figure 2:**
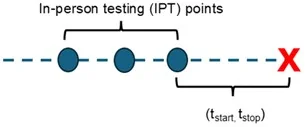
Illustration of the timeline used for the survival analysis. Biomarker measurements (dots) are collected at multiple testing occasions, IPTs, and used to estimate individual-level random effects. To avoid bias from using future information, the start of follow-up (t_start_) is defined as the age at the last biomarker measurement. This ensures that all data used for prediction precedes or coincides with the start of the survival model. The end of follow-up (t_stop_) is defined as the age at death or censoring.

The timescale of the survival model is age. The stopping time *t_stop_* is straightforward: it is defined as age at death or, for censored individuals, age at end of follow-up. Defining the starting time, *t_start_*, is more nuanced. When estimating the random effects from the mixed models, we aimed to maximize the number of repeated measurements used, as more data improves the stability of these estimates. Although random effects can technically be estimated from a single measurement, fewer repeats lead to greater shrinkage toward the population means. If *t_start_* were defined as age at first IPT, this would introduce bias: the random effects used as predictors at *t_start_* would be informed by biomarker measurements collected after that point in time. To avoid this kind of look-ahead bias, we instead define *t_start_* as age at the last IPT. This ensures that all biomarker data used to estimate random effects precede or coincide with the time at which they are used for prediction. See Figure 2 for a visual representation of this setup.

### Evaluation and comparisons

Predictors are ideally validated in an external dataset. For a predictor based on repeated measurements, this is often not possible: it requires a dataset in which the same variables are measured repeatedly within individuals. We did not have access to such a dataset to externally validate our weighted PoA.

A direct comparison between the original PoA and our weighted version is impossible for a similar reason: not all variables from the Dunedin data underlying the original PoA are available in the SATSA data, and vice versa. As a workaround, we created an unweighted PoA on the SATSA data ourselves by considering the same 10 variables as used for the wPoA. While the original PoA is based on individual-specific slopes, we use the corresponding random slopes, analogous to the wPoA. These carry the same information but are more convenient in the presence of non-linear modeling of the population trajectories. In the remainder of this manuscript, this predictor will be referred to as the unweighted PoA (uPoA).

We first examined to what extent the uPoA and wPoA correlate with established predictors of biological age. We compared uPoA and wPoA with DunedinPACE,^4^ GrimAge,^17^ PhenoAge,^18^ physiological age (PA),^19^ the Frailty Index (FI),^20^ and Functional Aging Index (FAI).^21^ Details of the use of these predictors in the SATSA data can be found in Li et al.^19^ PA was originally fitted on the SATSA data; FAI was partly. For PhenoAge and GrimAge, we used the new principal components-based versions of these clocks. For all predictors but DunedinPACE (which is not a biological age predictor but a methylation-based predictor of the original PoA), we regressed out the effect of chronological age to obtain the chronological age-independent ‘age accelerations.’

We also examined the association of uPoA and wPoA with time-to-event outcomes in SATSA, including mortality, cardiovascular disease (CVD), diabetes, and dementia. However, because the weights underlying wPoA are estimated within the same dataset, such comparisons may be affected by overfitting, potentially leading to optimistic estimates of association when evaluating the wPoA. To mitigate this concern, and in the absence of an external dataset, we conducted an additional analysis using externally derived survival-based weights. In this variant, denoted wPoA*, biomarkers were weighted according to previously published associations with all-cause mortality in other studies (Supplementary Note 3). This approach allows us to assess the added value of mortality-informed weighting while reducing the risk of overfitting inherent to internally estimated weights. Due to high correlations between FVC, FEV1, and FVC/FEV1 and the lack of suitable external weights, only FVC was retained in this analysis. Hemoglobin was excluded due to limited availability of external data. We then evaluated the association of wPoA* with the same time-to-event outcomes. Results are presented in Table 2 alongside those obtained using the corresponding unweighted PoA (uPoA*) based on the same biomarkers. Detailed definitions of the outcomes, the number of events observed, and the total follow-up time are provided in Supplementary Information, Note 4.

**Table 1:** Sample characteristics and biomarker summary statistics. Values are calculated across all 745 participants included in the analysis. Biomarker values represent the mean across all available testing occasions per individual; standard deviations are shown in parentheses. Follow-up time refers to the period between *t_start_* and *t_stop_*. Motor factors flexibility and balance do not have a unit in the table as they are the result of a factor analysis on multiple items related to functional ability; see Finkel et al.^22^ for more details.

Characteristic	Value
Total number of participants	745
Number of deaths during follow-up (%)	572 (76.8 of cohort)
Sex (% women) at first IPT (%)	61.5
Mean number of IPT	4.8 (*SD* = 2.7)
Median follow-up time (first IPT–*t_stop_*)	22.7 years (IQR: 15.5–30.4)
Median follow-up time in the survival model (last IPT–*t_stop_*)	6.5 years (IQR: 2.5–10.9)
Mean age at first IPT	62.3 (*SD* = 8.5)
Mean age at last IPT	77.1 (*SD* = 8.8)
Waist-hip ratio	0.9 (0.07)
Grip strength (kg)	26.3 (1.9)
MMSE7 (0–30 point scale)	27.3 (3.2)
Flexibility	2.3 (0.8)
Balance	11.8 (3.8)
FEV1 (L)	2.1 (0.7)
FVC (L)	2.4 (0.9)
FEV1/FVC	0.9 (0.1)
Hemoglobin (g/L)	139.8 (14.1)
Blood sugar (mmol/L)	5.2 (1.6)

**Table 2:** Hazard ratios (HRs) for associations between the weighted Pace of Aging (wPoA), unweighted Pace of Aging (uPoA), externally weighted Pace of Aging (wPoA*), and the unweighted Pace of Aging based on the same reduced number of biomarkers as wPoA* (7 instead of 10) due to the lack of external information (uPoA*) and four age-related outcomes: cardiovascular disease (CVD), diabetes, dementia, and all-cause mortality. The scale of the Cox model is age, and all models were adjusted for sex. *p*-values are shown in parentheses.

	CVD	Diabetes	Dementia	All-cause mortality
wPoA	1.49 (<0.001)	1.27 (0.154)	1.44 (0.002)	1.50 (<0.001)
PoA	1.35 (0.002)	1.16 (0.38)	1.53 (<0.001)	1.42 (<0.001)
wPoA*	1.34 (0.002)	1.21 (0.214)	1.67 (0.010)	1.41 (<0.001)
uPoA*	1.29 (0.009)	1.12 (0.492)	1.61 (<0.001)	1.40 (<0.001)

## Results

### Sample characteristics

Table 1 shows the characteristics of the study sample. The mean age at baseline was 62.3% and 61.5% of participants were women. By the last wave, the mean age had increased to 77.1. By the end of follow-up, 76.8% of the sample had died. The median follow-up time was 22.7 years. The ten biomarkers included in the weighted PoA construction are also listed in Table 1. Details on the biomarkers can be found in Supplementary Information, Note 1.

### Descriptives weighted Pace of Aging

**Figure 3:**
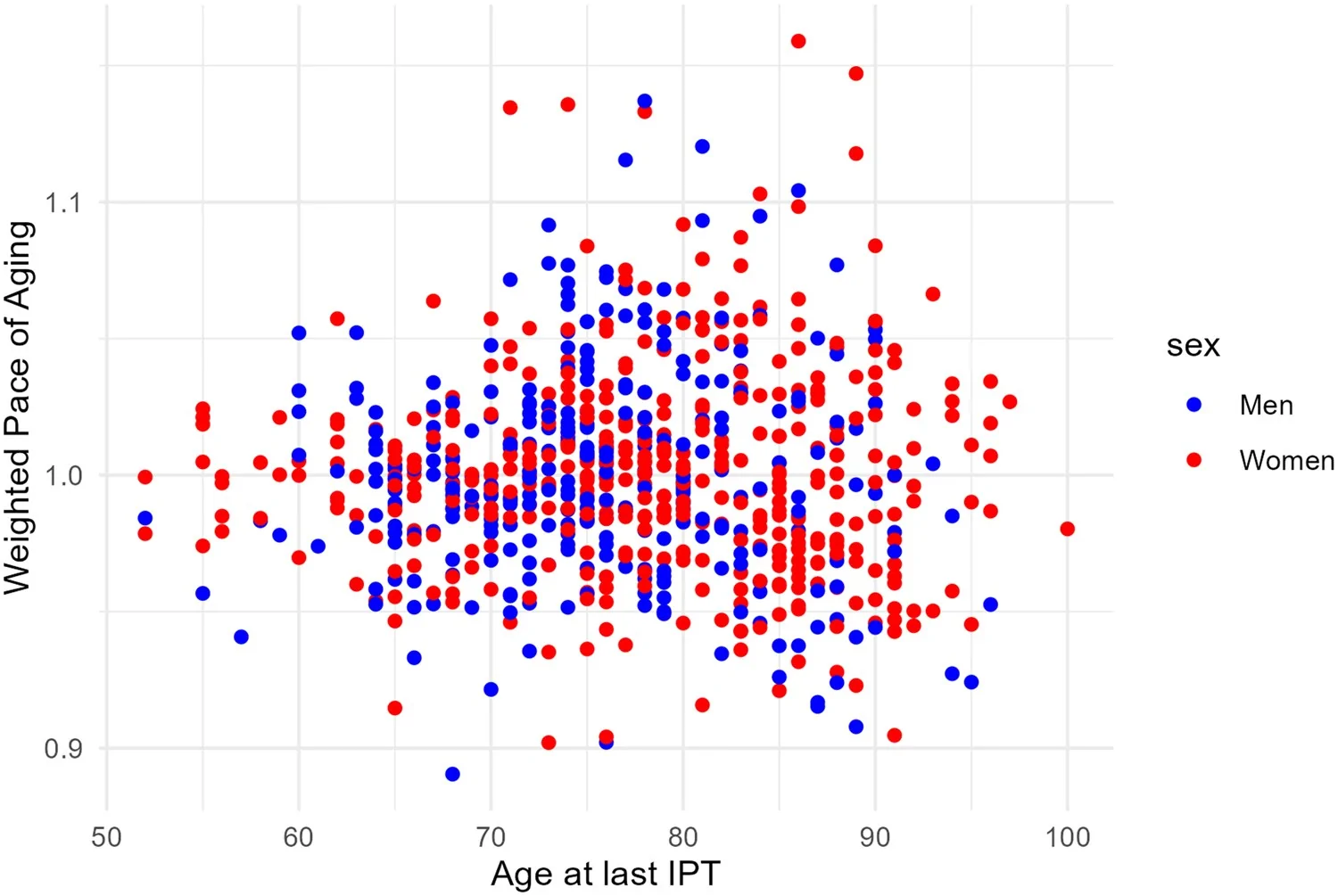
Weighted Pace of Aging (y-axis) against chronological age at last IPT (x-axis) in SATSA. The gray line represents the fitted linear regression line (slope coefficient = −4.43e-6, *p* = .978).

We calculated the wPoA for the 745 individuals in the final sample. A plot of wPoA against chronological age (at the last IPT, see Figure 2) can be found in Figure 3. The mean wPoA in the cohort is (per definition) 1. The standard deviation is 0.04; the range is 0.89–1.16. The gray line represents the linear regression of wPoA on chronological age: there is no evidence of an association between age acceleration and chronological age (slope coefficient = −4.43e-6, *p *= .978). Men and women exhibit a similar pattern and similar spread. The specific weights of each biomarker in wPoA can be found in Table S2 in Supplementary Information, Note 3.

### Association with aging-related health outcomes

Table 2 shows the association of wPoA, uPoA, the externally weighted Pace of Aging (wPoA*), and its unweighted counterpart (uPoA*) with time-to-event outcomes, including cardiovascular disease, diabetes, dementia, and all-cause mortality. The table reports hazard ratios from Cox proportional hazards models for the scaled and centered PoA measures.

All versions of the Pace of Aging were positively associated with incident cardiovascular disease, dementia, and all-cause mortality, with weaker and non-significant associations for diabetes. Weighted versions presented, in general, larger hazard ratios than unweighted versions, but the differences were small. For cardiovascular disease and all-cause mortality, wPoA showed slightly stronger associations than uPoA, whereas for dementia the unweighted version showed a somewhat stronger association than the weighted version. As expected, the associations for wPoA*, except for the association with dementia, were slightly attenuated compared with wPoA, supporting the idea that external weighting may help reduce overfitting.

### Correlation with established biological aging indices

**Figure 4:**
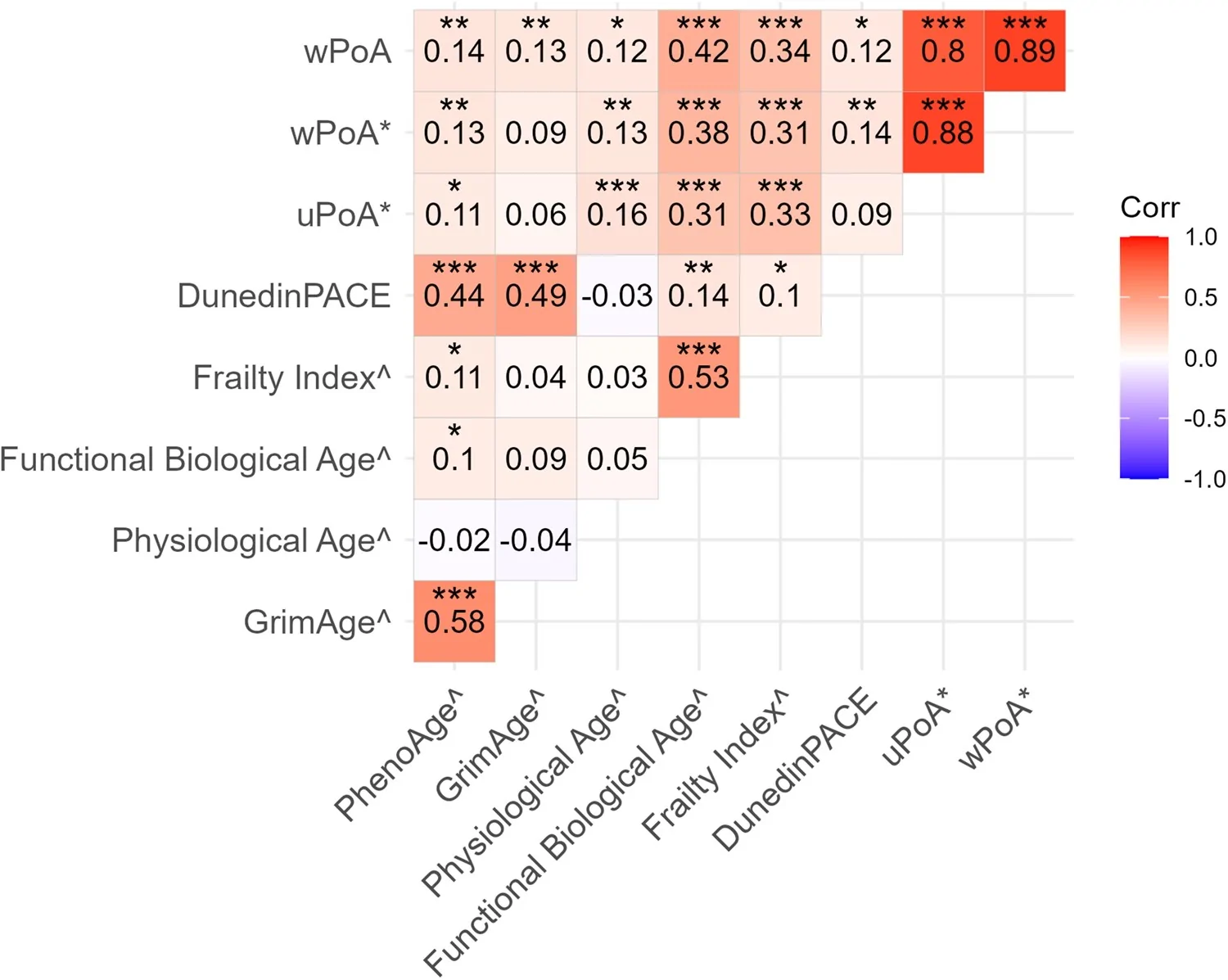
Correlation plot of biological age and aging rate predictors. Complete measurements from 425 individuals were considered. Symbols ^ for the predictors indicate that chronological age was regressed out of the prediction (i.e., these are the so-called ‘age accelerations’). The asterisks in the square boxes of the plot indicate the significance of the correlation coefficients (∗ = *p* ≤ .05. ∗∗ = *p* ≤ .01. ∗∗∗ = *p* ≤ .001).

Figure 4 contains a correlation plot of the correlation (Pearson’s correlation coefficient) between various (chronological age-adjusted) biological ages, paces of aging, and our novel weighted Pace of Aging (wPoA) and the version based on external weights wPoA*. The correlation was calculated based on one measurement per individual, namely at the last IPT for which data was available.

It can be seen that wPoA is most strongly correlated with the unweighted PoA and the wPoA*. This makes sense: these predictors are based on the same data and the same underlying set of markers. After that, the wPoA and wPoA* are most strongly correlated with the Functional Aging Index and the Frailty Index. The wPoA is significantly correlated with (the chronological-age-independent parts of) GrimAge and DunedinPACE, while the unweighted PoA is not, although the differences in correlation coefficients are small. The trend is similar for wPoA*, although its correlation with GrimAge is weaker. In general, the unweighted PoA shows weaker correlations with other aging, except for (the chronological-age-independent parts of) physiological age.

## Discussion

In this study, we developed a wPoA predictor using longitudinal data from SATSA, a cohort of older adults followed for three decades. Our approach builds on the conceptual foundation of the original PoA model,^3^ and incorporates methodological innovations that improve flexibility and applicability to aging populations with diverse and potentially noninformative biomarkers. We examined the association of the wPoA with other biomarkers of aging. As a fully independent external validation was not feasible, we constructed an externally weighted version of the wPoA, the wPoA*, to enable a less biased evaluation of its performance in predicting health outcomes. A limitation of this approach is that the external weights are derived from hazard ratios reported in the literature and therefore differ in interpretation and scale from the coefficients in our model. As such, wPoA* should be viewed as a sensitivity analysis rather than a fully validated external predictor. Future research should aim to evaluate the external validity of wPoA in independent longitudinal cohorts with comparable repeated biomarker measurements.

An advantage of the weighted approach we adopted is that it allows each biomarker’s contribution to the aging rate to be determined empirically, rather than assumed a priori. Although all ten biomarkers we included are known to be informative of aging and/or disease, this need not generally be the case. The weighting may be particularly useful in settings with a larger number of candidate markers, where their relevance is uncertain. A mortality-informed weighting approach can then downweigh less informative slopes, resulting in a more reliable measure than simply summing all slopes.

Compared to an unweighted PoA, the new wPoA and the externally weighted wPoA* seem to show stronger associations with time to CVD and time to diabetes. Although the effect is not significant for the latter outcome. Interestingly, the unweighted PoA had a stronger association with time-to-dementia than the weighted PoA. When considering external weights, this is no longer the case. However, this behavior illustrates an interesting point. uPoA better predicts time-to-dementia than wPoA can, which is explained by the role of MMSE, a cognitive measure that is highly predictive of dementia but not as strongly predictive of mortality. In the unweighted PoA, MMSE contributes to the composite aging rate regardless of its association with mortality. In contrast, the wPoA did not assign large coefficients to the random slope of MMSE due to its limited prognostic value for mortality compared to the other included predictors, thereby reducing its predictive power for prospective dementia.

Our correlation analyses showed that wPoA is moderately correlated with several well-established biological aging measures. Specifically, we found positive associations with methylation-based predictors such as PhenoAge and somewhat stronger correlations with lower-dimensional phenotypic indicators like the Functional Aging Index (partly based on the same underlying markers and data as wPoA) and the FI. However, all correlations were modest in magnitude. This is consistent with the previous findings,^19^ who also found limited overlap among various biological age measures in SATSA. This suggests that different predictors may tap into different aspects of the multidimensional aging process.

Due to differences in biomarker availability between studies, we could not directly apply or compare our wPoA to the original Dunedin PoA. This illustrates a general limitation of repeated measurement-based predictors: application in other datasets would require repeated measurements of the same variables, which are generally not available. This limitation partly motivated the development of single-timepoint methylation-based surrogates like DunedinPoAm and DunedinPACE,^4^^,^^5^ which were trained to emulate the Dunedin PoA. Nonetheless, improvements to the underlying PoA (such as the weighting approach that we propose) likely also improve the performance of downstream single-timepoint predictors trained on it.

Another strength of our approach is its suitability for older populations. The original PoA was trained in a relatively young cohort, where relationships between markers and age were mostly linear and in which mortality was too rare to serve as an outcome. In contrast, SATSA participants were 50 and older at baseline; some included markers showed a clear nonlinear relation with age, and a substantial proportion had died by the end of follow-up. This allowed us to weigh biomarkers based on their association with time-to-mortality. We allowed for non-linear age trajectories by incorporating splines for age in the mixed models as previously proposed in a similar setting.^11^

Biomarker selection in older adults comes with its own challenges, such as a reversing effect in the oldest old and widespread medication use, which can affect lipid markers. We addressed these challenges through careful model design and exclusion criteria. Not all modeling adjustments may be necessary or optimal in younger populations, and other adjustments may be possible as well, but some adjustments are critical for capturing heterogeneity in older cohorts.

A related contribution,^23^ demonstrated that changes in epigenetic clocks over time can predict survival independent of baseline biological age. Although their work used established single-timepoint methylation-based biological aging clocks instead of developing a new aging rate predictor based on repeated measurements, their work also illustrates the growing interest in changes in biological aging over time. In our analysis, we chose to prioritize maximizing the number of repeated measurements per individual, rather than maximizing the overall follow-up time. This, however, comes at the cost of having less follow-up time. Different choices could be made here: for example, an appealing alternative approach might resemble dynamic landmarking, where models are fitted at several predefined time points among individuals still at risk.^24^ Applied to PoA, this could involve fitting mixed-effects models at, e.g., 5-year age intervals for all individuals still at risk at those time points, using only biomarker data from the preceding 10 years. While methodologically appealing, this method was not feasible with the SATSA dataset due to its limited sample size and average number of repeated measurements.

To create the wPoA, we used a two-step approach:^25^^,^^26^ we first fitted mixed-effects models to estimate individual-level random effects and then used these as predictors in a survival model. While this is straightforward and interpretable, this approach may suffer from bias and inefficiency under some conditions.^27^^,^^28^ Methodologically more advanced methods exist: in particular, joint models that estimate the longitudinal and survival components simultaneously might be appealing.^27^^,^^29^ However, joint models can be challenging to use because they are computationally intensive to fit. As an additional complication, little research has been done on the use of joint models in clustered data (such as SATSA),^30^ and the methods that do exist have not yet been extended to the AFT model framework. We plan to further develop joint models for aging pace predictors in future work.

In sum, our proposed wPoA model offers a more flexible and data-driven framework for estimating aging rates in older adults. It may serve as a more robust foundation for future work on biological aging, especially in settings with diverse biomarker sets or limited prior knowledge of marker relevance.

## Supplementary Material

glag205_Supplementary_Data

## Data Availability

The SATSA cohort has been archived through the NACDA Program on Aging (www.icpsr.umich.edu/icpsrweb/ICPSR/studies/3843), and detailed information on the study can be found on the Maelstrom Research platform (www.maelstrom-research.org/mica/individual-study/satsa). Data access can be applied for through the NEAR platform (www.near-aging.se/studies-included/application/).
