# Supplementary material for: A generalized approach for estimating the Pace of Aging
Source: J Gerontol A Biol Sci Med Sci. 2026 Aug 20;81(10):glag205. doi: 10.1093/gerona/glag205 (PMC13600683; doi:10.1093/gerona/glag205)
Supplement: Supplementary file 1 [file glag205_supplementary_data.pdf]

A generalized approach for estimating the pace of aging

Marije H. Sluiskes, PhD[1,2], Joris Deelen, PhD[1], Hein Putter, PhD[1], Sara Hägg, PhD[2], and Mar Rodriguez-Girondo, PhD*[1]

[1]Biomedical Data Sciences, Leiden University Medical Center, Leiden, The Netherlands; [2]Medical Epidemiology and Biostatistics, Karolinska Institutet, Stockholm, Sweden

* Address correspondence to: Mar Rodriguez-Girondo, PhD. E-mail: m.rodriguez_girondo@lumc.nl

Contents of the supplementary materials:

Supplementary Information Note 1: information on the biomarkers in SATSA.

Supplementary Information Note 2: plots of the fixed effects of the mixed-effects models fitted on SATSA biomarkers.

Supplementary Information Note 3: information on the construction of the wPoA* the weighted pace of Aging based on external weights.

Supplementary Information Note 4: definition of the health outcomes considered in Table 2.

Supplementary Information: Note 1

A generalized approach for estimating the pace of aging

Marije H. Sluiskes, PhD[1,2], Joris Deelen, PhD[1], Hein Putter, PhD[1], Sara Hägg, PhD[2], and Mar Rodriguez-Girondo, PhD*[1]

(m.rodriguez_girondo@lumc.nl)

[1]*Biomedical Data Sciences, Leiden University Medical Center, Leiden, The Netherlands;* [2]*Medical Epidemiology and Biostatistics, Karolinska Institutet, Stockholm, Sweden*

This Note contains information on the biomarkers in SATSA, both the 10 markers included in the weighted Pace of Aging as well as the markers that were (due to various reasons, discussed in Table S1 below) not included. In Table S1, variable definitions and reasons for exclusion are given. The figures that follow show the spaghetti plots.

Table S1. Overview of biomarkers in SATSA that were considered for the weighted Pace of Aging.

**Biomarker**	**Abbreviation (in plots)**	**Median number of rep. meas.**	**Incl. in wPoA?**	**Reason excl.***
ApoA1	apoa	4	No	C
ApoB	apob	4	No	C
Ratio ApoB/ApoA1	ratioba	4	No	C

B12	b12	0	No	A
Blood sugar	blsk	4	Yes	
BMI	bmi	4	No	B
s-CRP	crp	0	No	A
Grip strength	grip	4	Yes	
Hemoglobin	hb	4	Yes	
HDL	hdl	2	No	A
Cholesterol	kol	4	No	C
LDL	ldl	0	No	A
FEV1	lung_fev1	4	Yes	
FVC	lung_fvc	4	Yes	
FEV1/FVC	lung_fev1_fvc_ratio	4	Yes	
Mini mental state examination	mmse	4	Yes	
Balance	motor_bal	4	Yes	
Fine motor skills	motor_fine	3	No	A
Flexibility	motor_flex	4	Yes	
Ratio LDL/HDL	ratiolh	0	No	A
Free T4	t4	0	No	A
Triglycerides	tg	4	No	B
TSH	tsh		No	A
Waist-hip ratio	whr		Yes	

***Legend exclusion reasons:**

- A = median number of repeated measurements too low (< 4), due to some markers not being recorded in all in-person testing (IPT) occasions
- B = non-monotone relationship of marker with mortality over the lifespan
- C = unexpected relationship of marker with age (all lipids, likely due to lipid-altering medication)

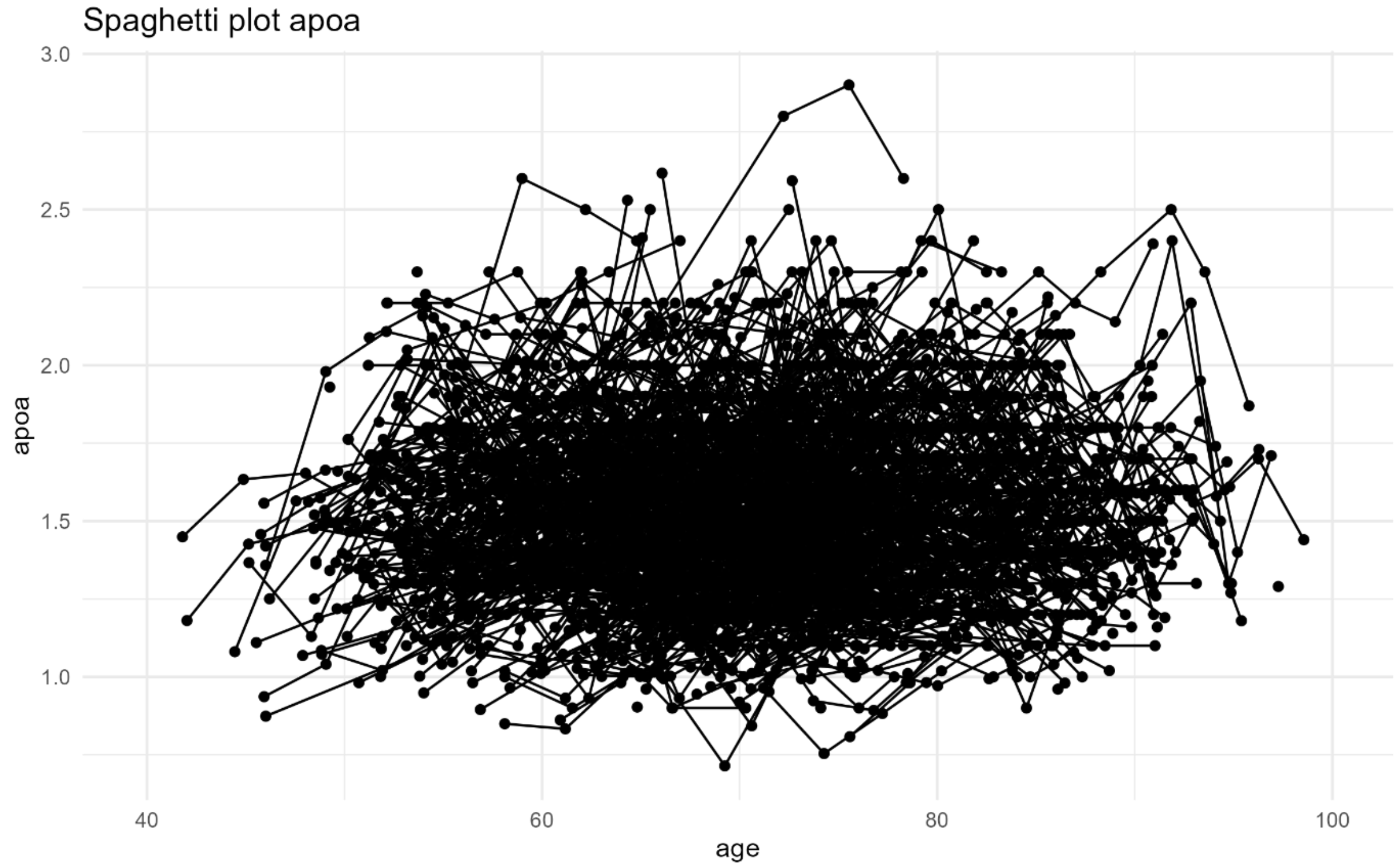
Spaghetti plot apoa
3.0
2.5
2.0
1.5
1.0
apoa
40
60
80
100
age

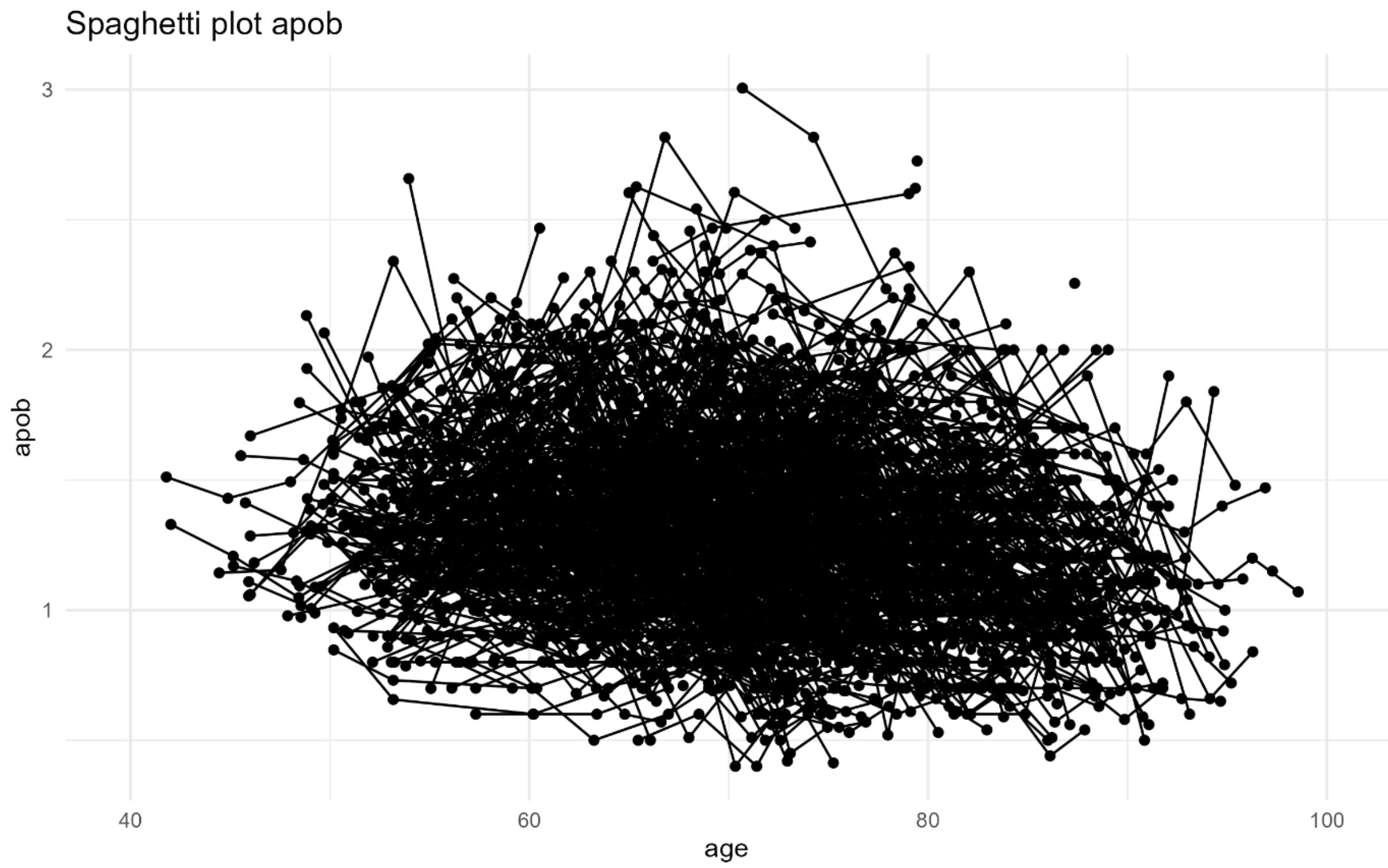
Spaghetti plot apob
3
2
1
apob
40
60
80
100
age

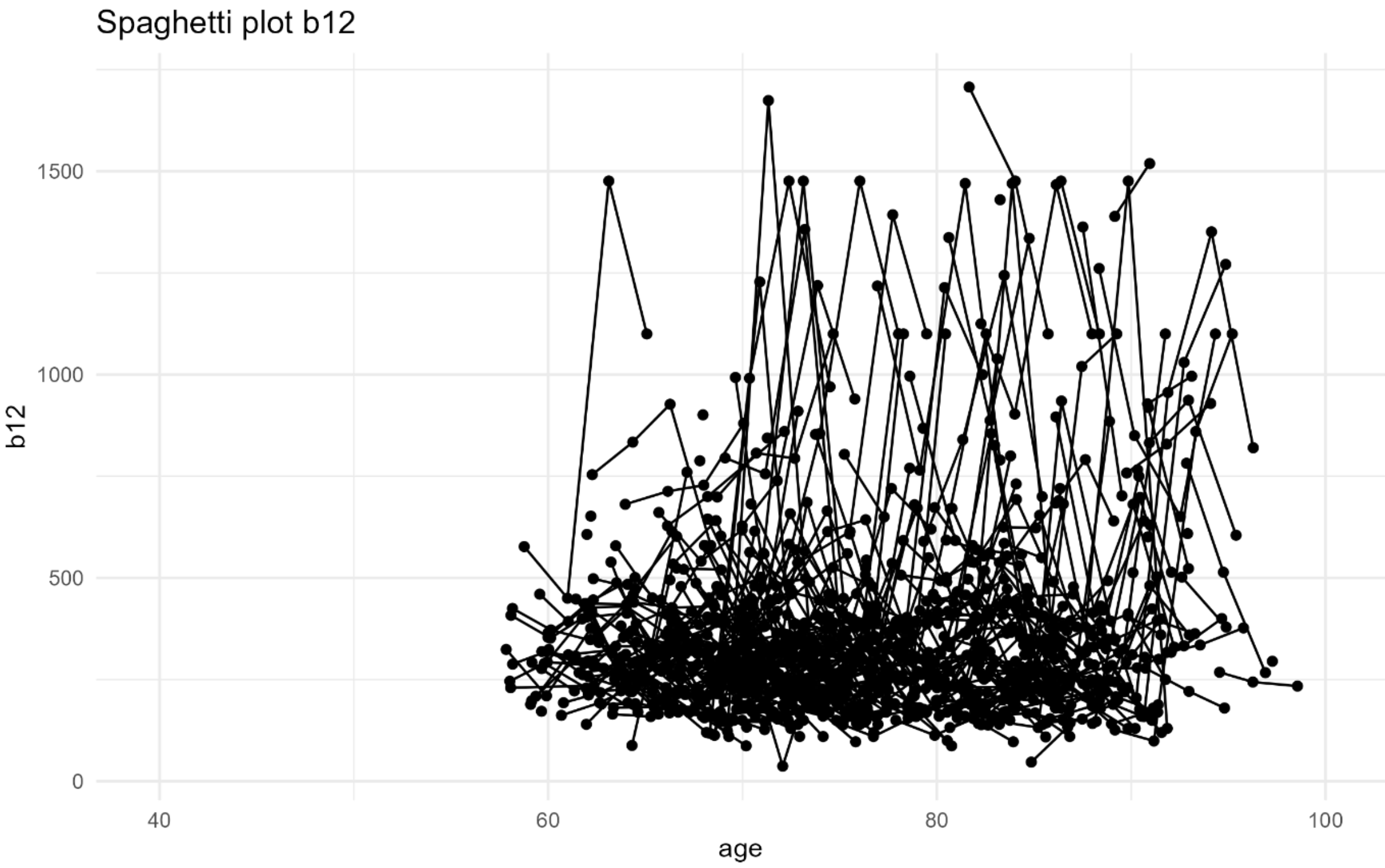
Spaghetti plot b12
b12
age
0
500
1000
1500
40
60
80
100

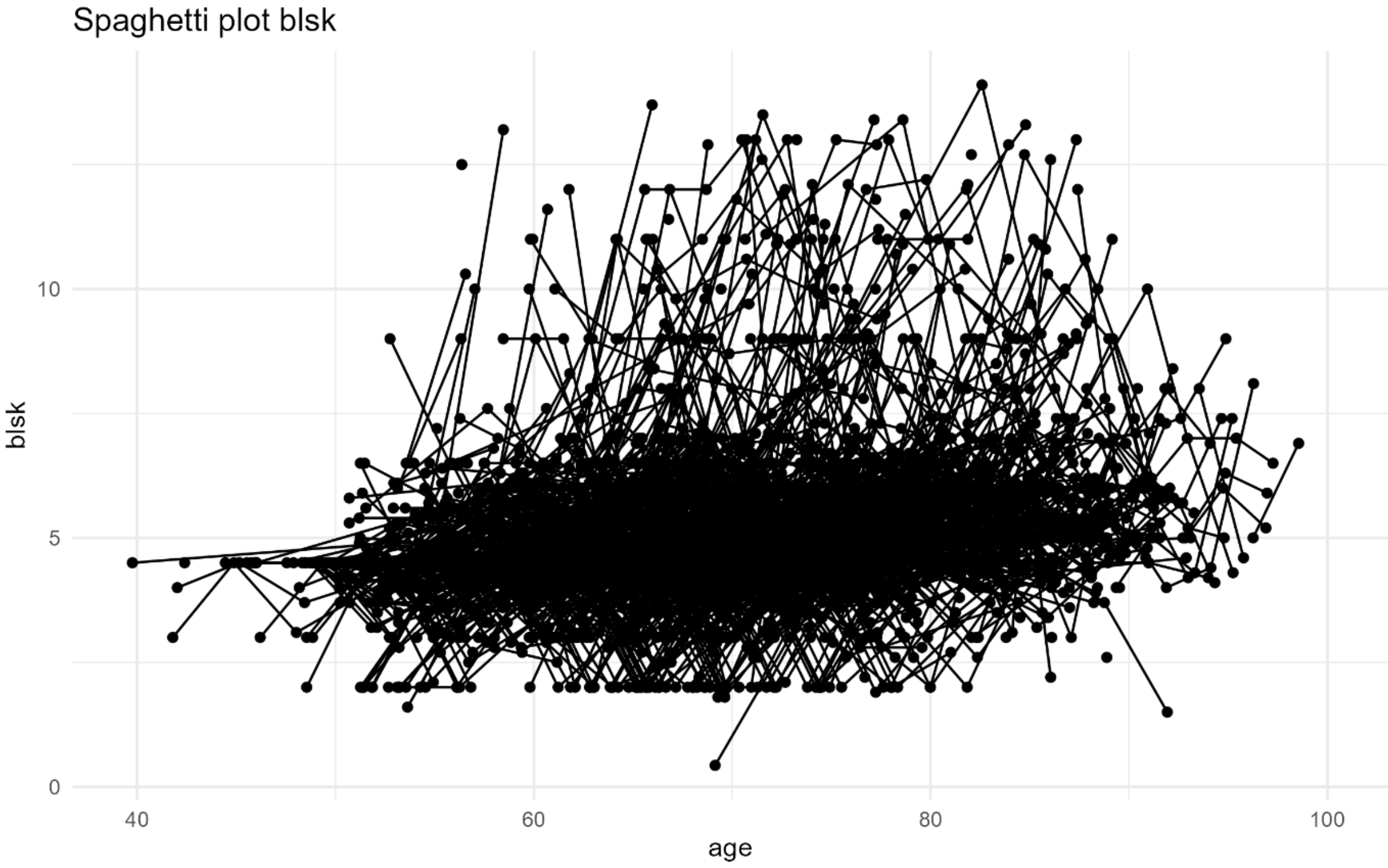
Spaghetti plot blsk
blsk
age
0
5
10
40
60
80
100

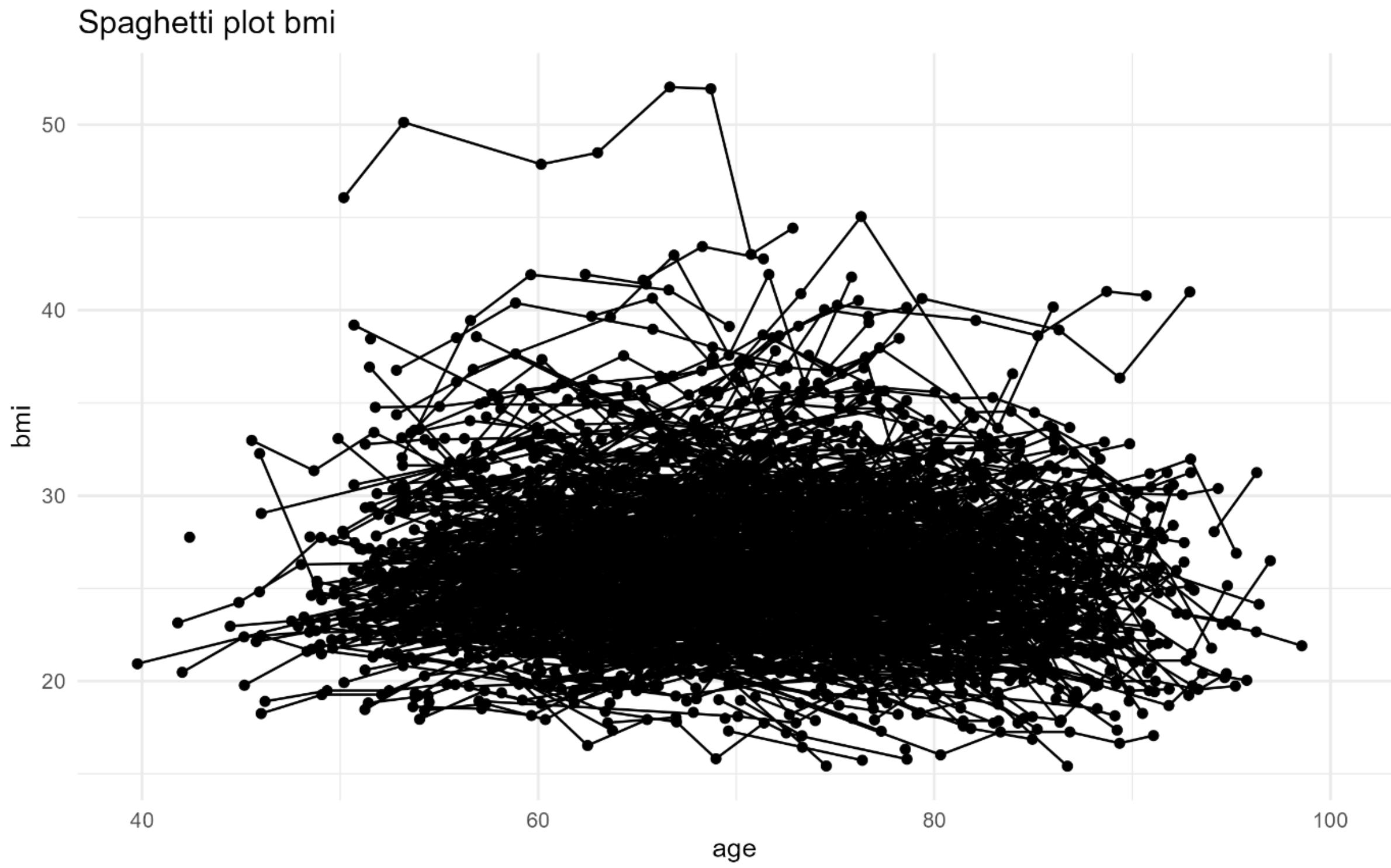
Spaghetti plot bmi
bmi
50
40
30
20
age
40
60
80
100

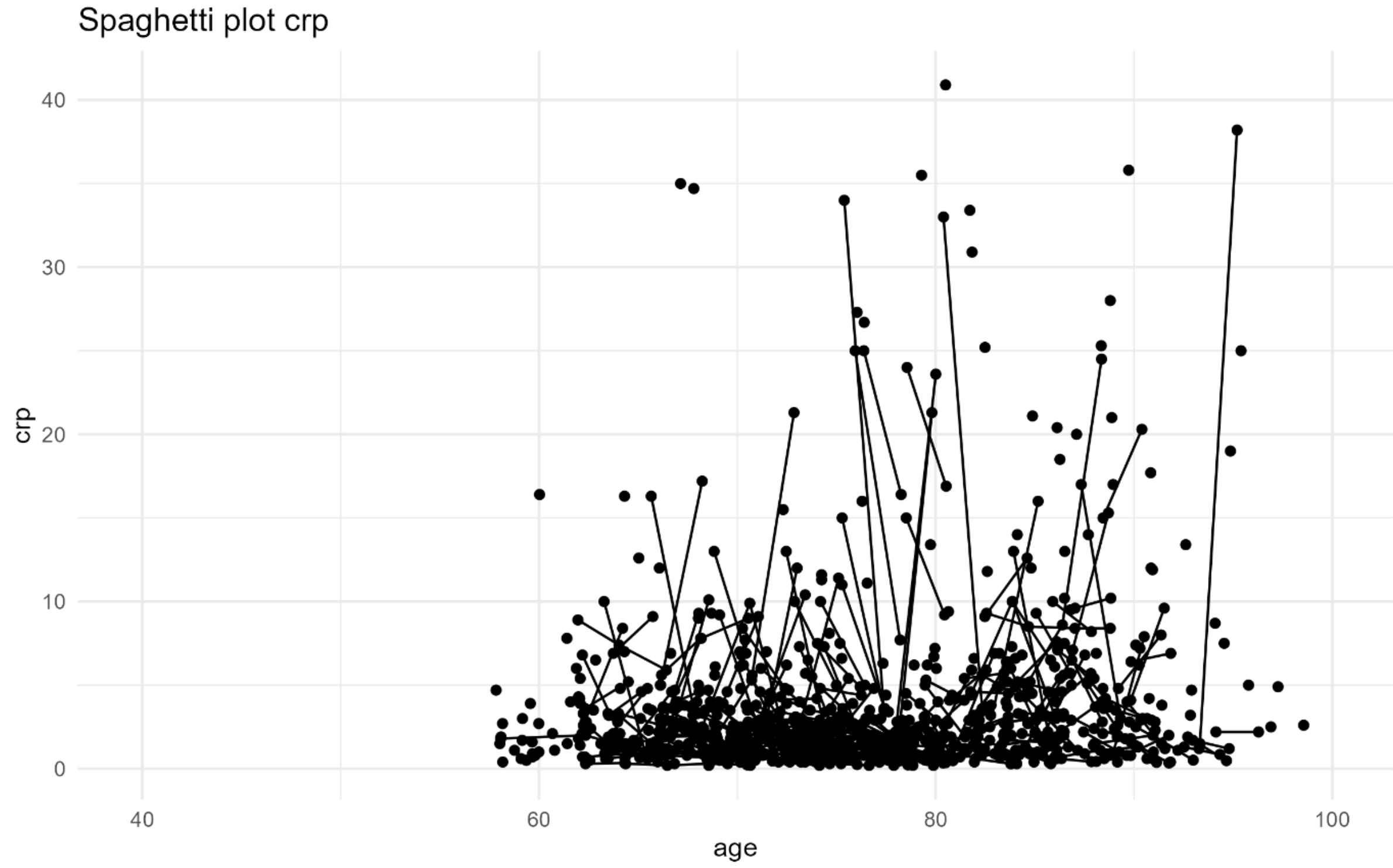
Spaghetti plot crp
crp
40
30
20
10
0
age
40
60
80
100

Spaghetti plot grip

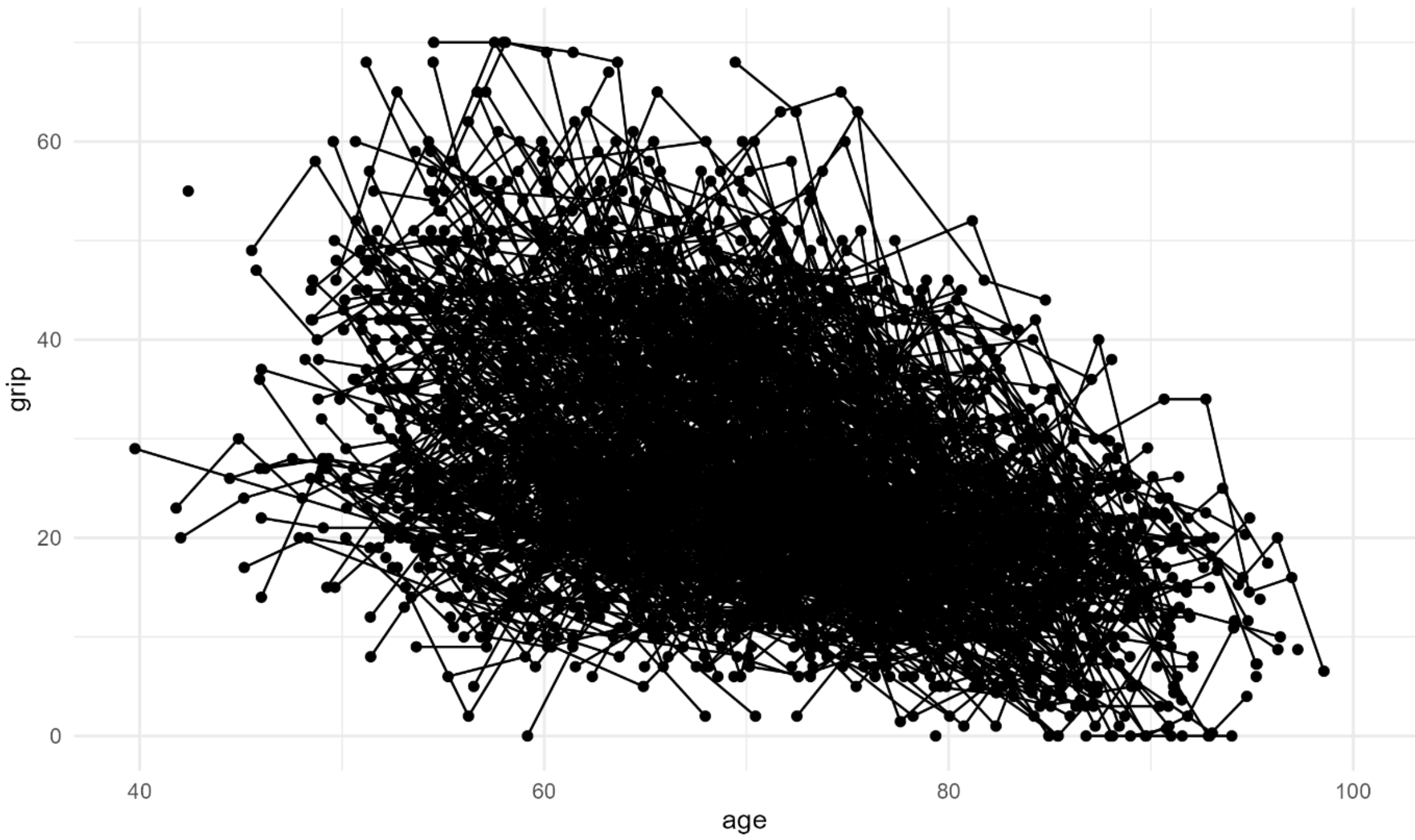

Spaghetti plot hb

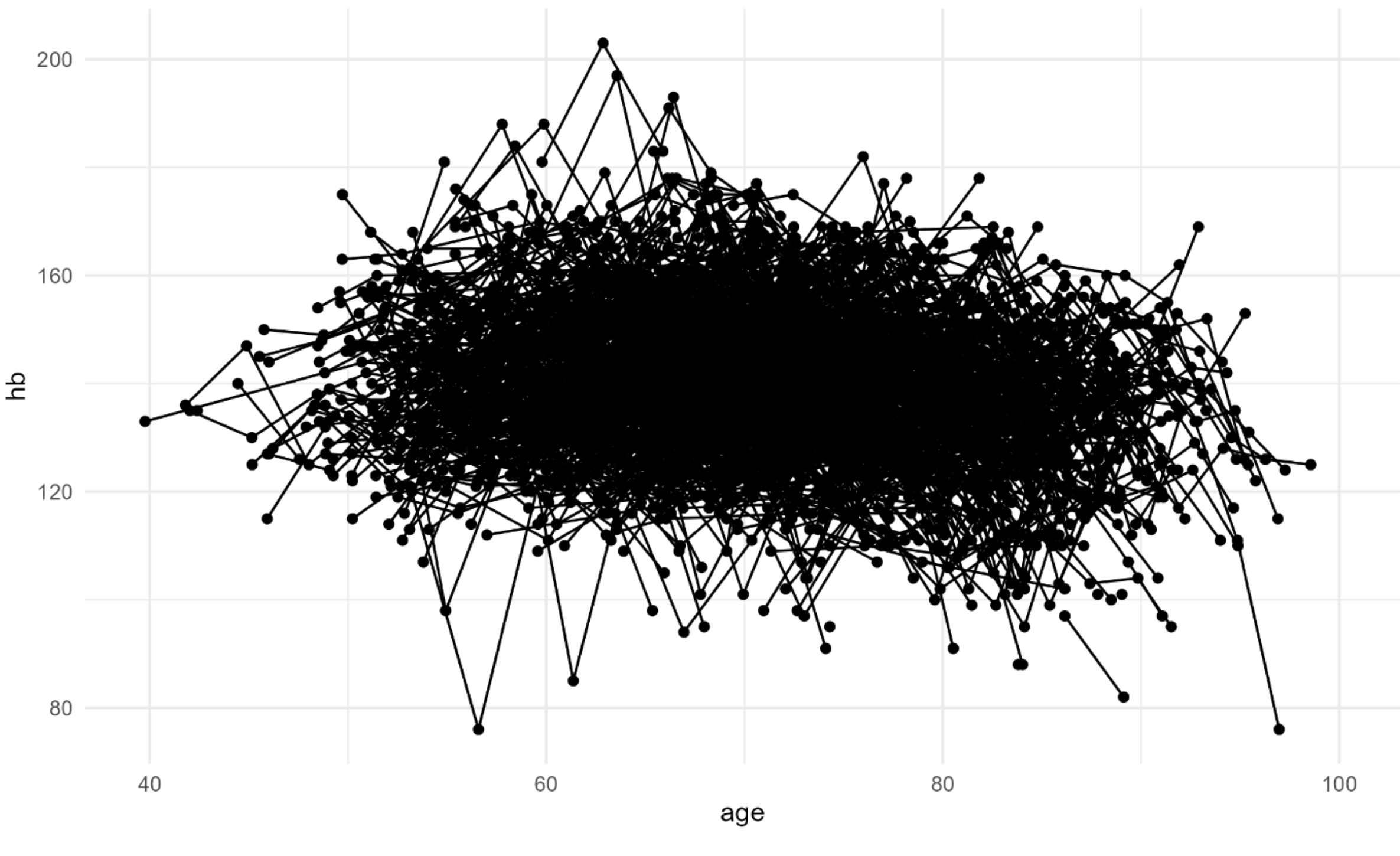

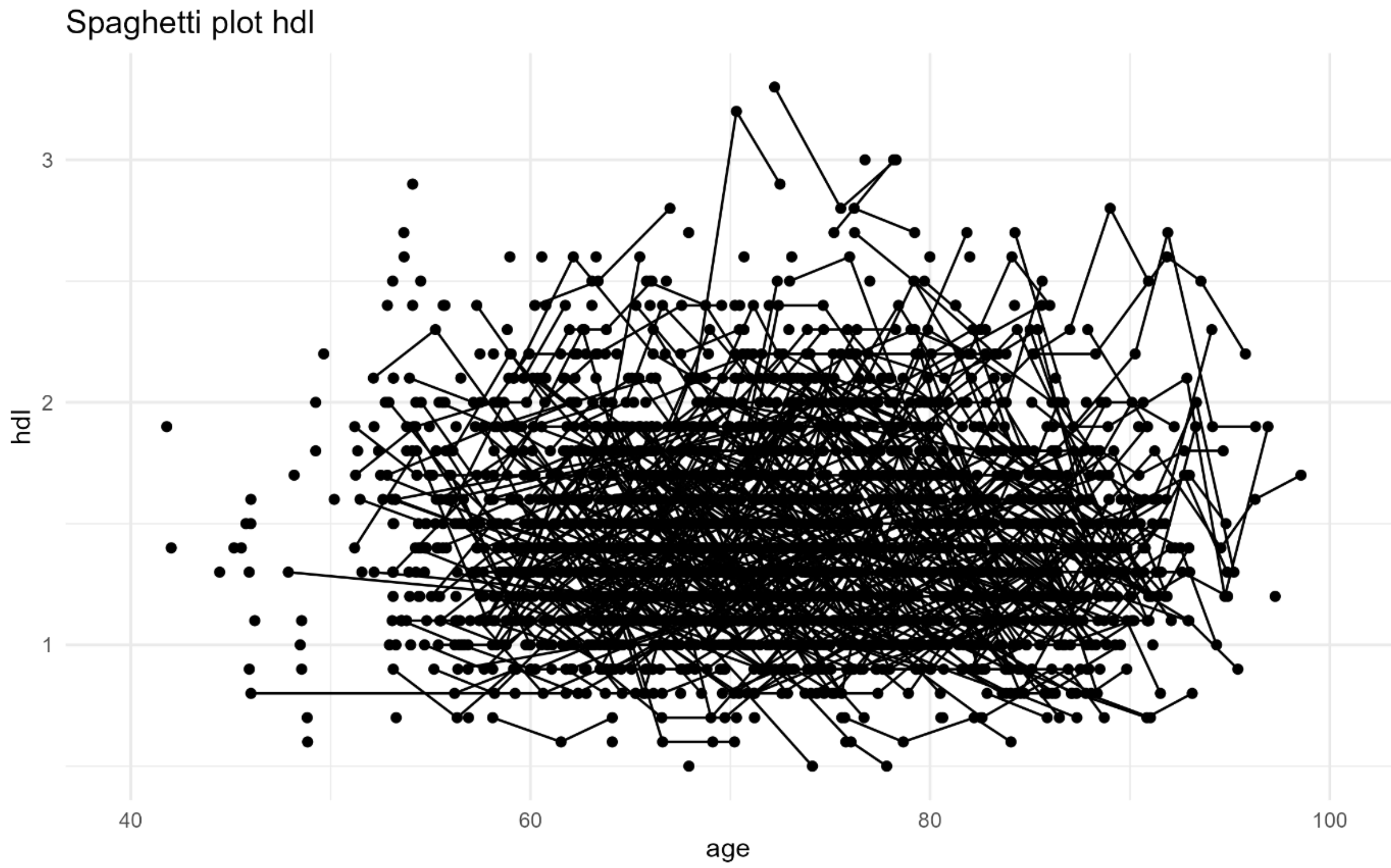
Spaghetti plot hdl
hdl
3
2
1
40
60
80
100
age

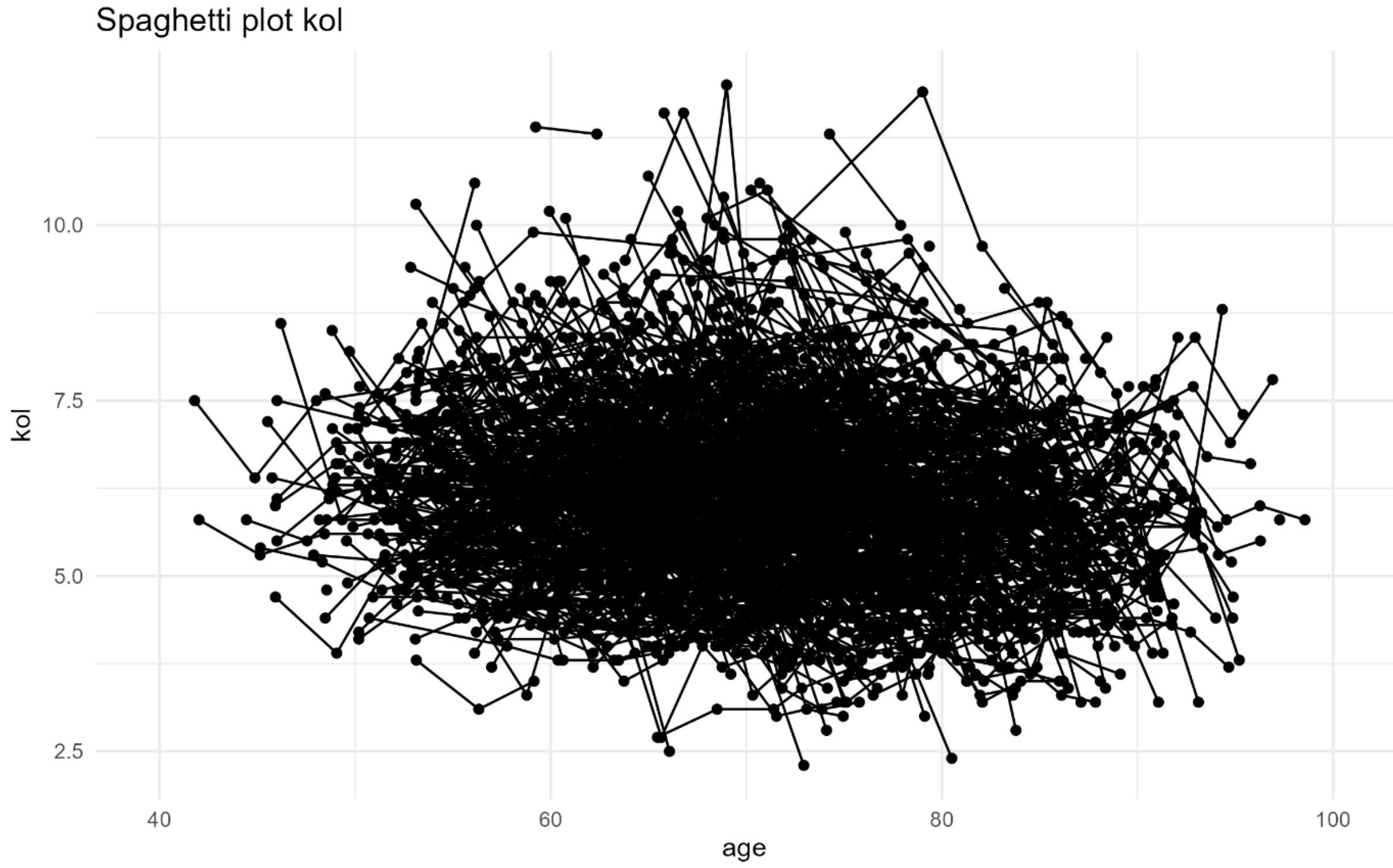
Spaghetti plot kol
kol
10.0
7.5
5.0
2.5
40
60
80
100
age

Spaghetti plot ldl

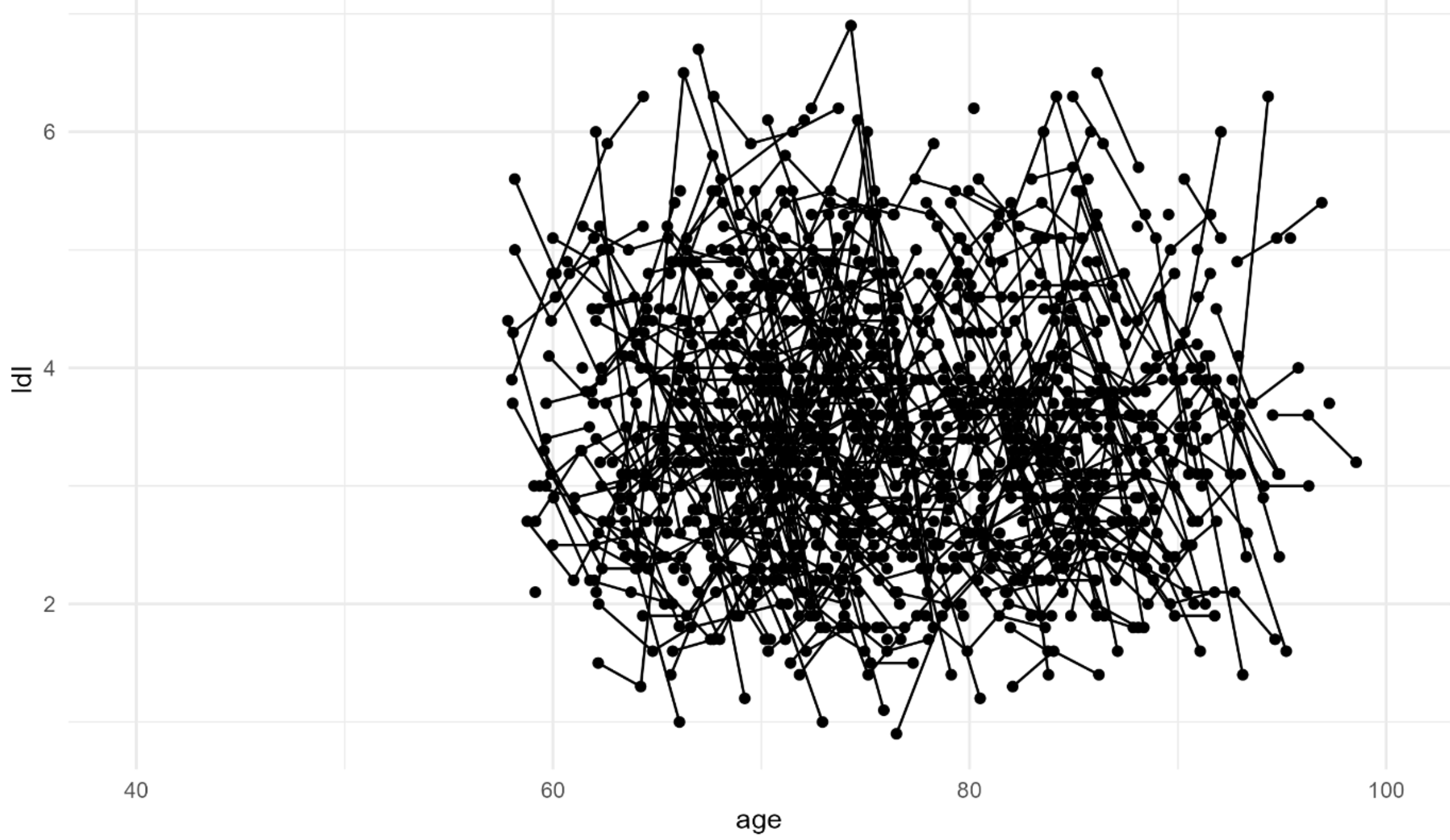

Spaghetti plot lung_fev1

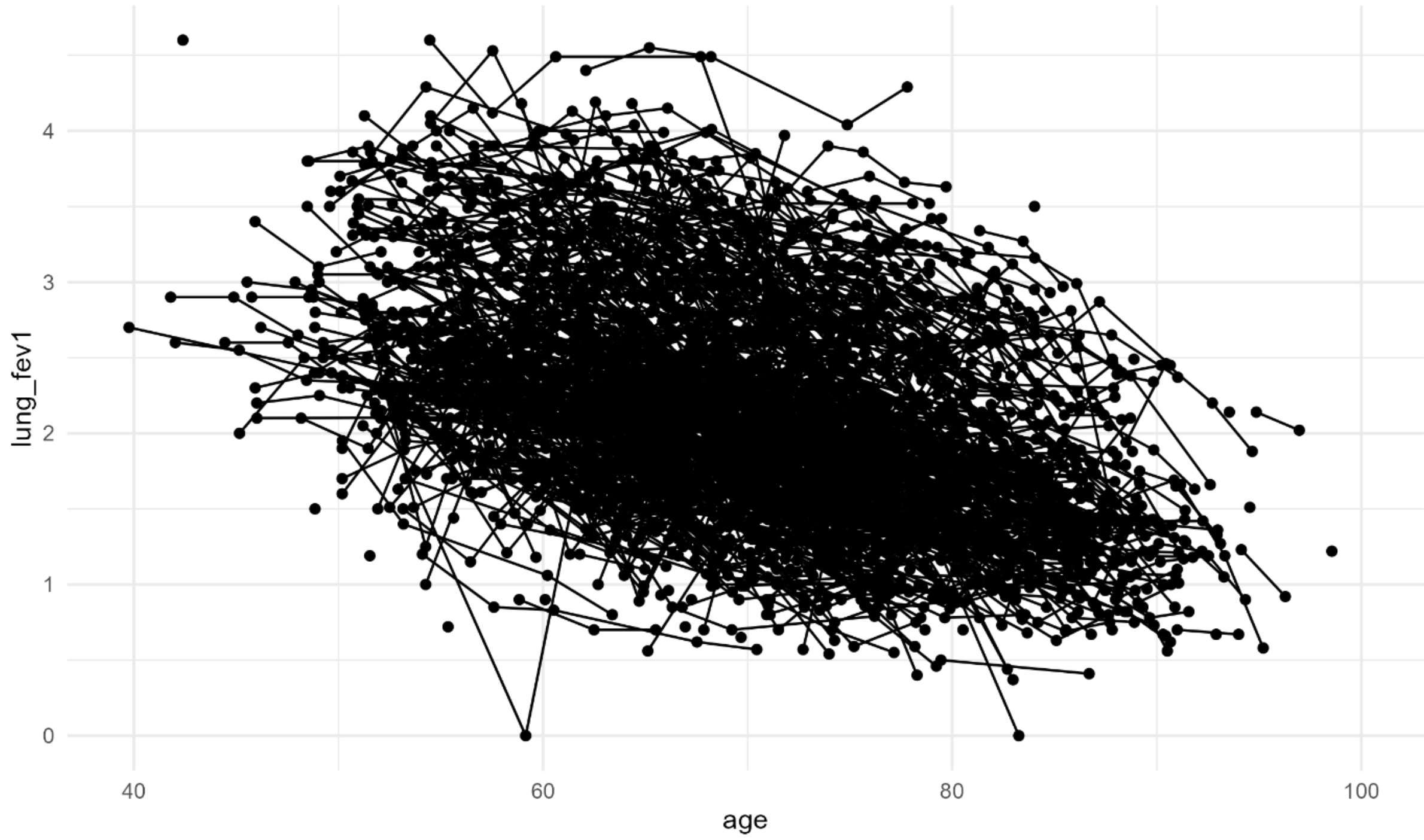

Spaghetti plot lung_fev1_fvc_ratio

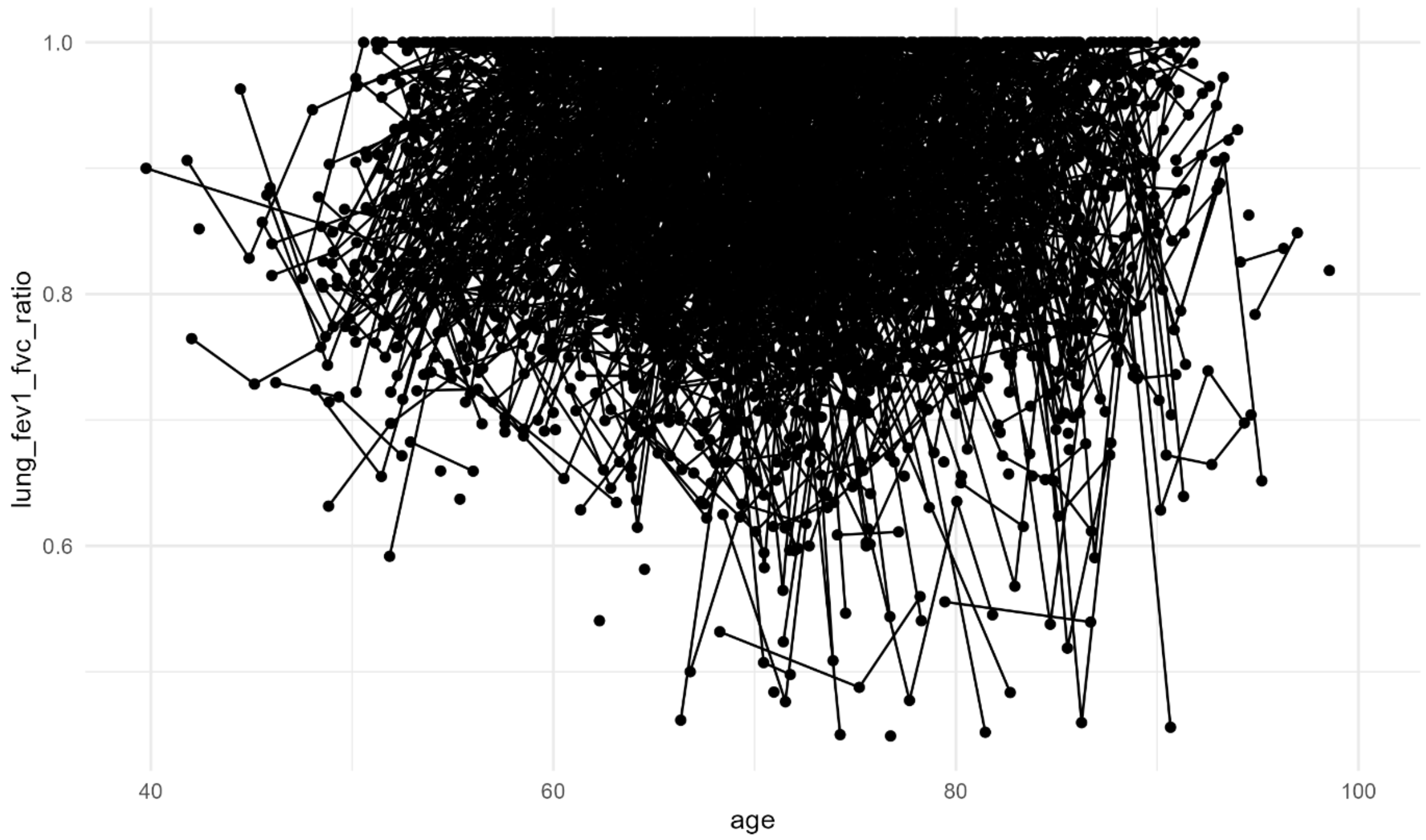

Spaghetti plot lung_fvc

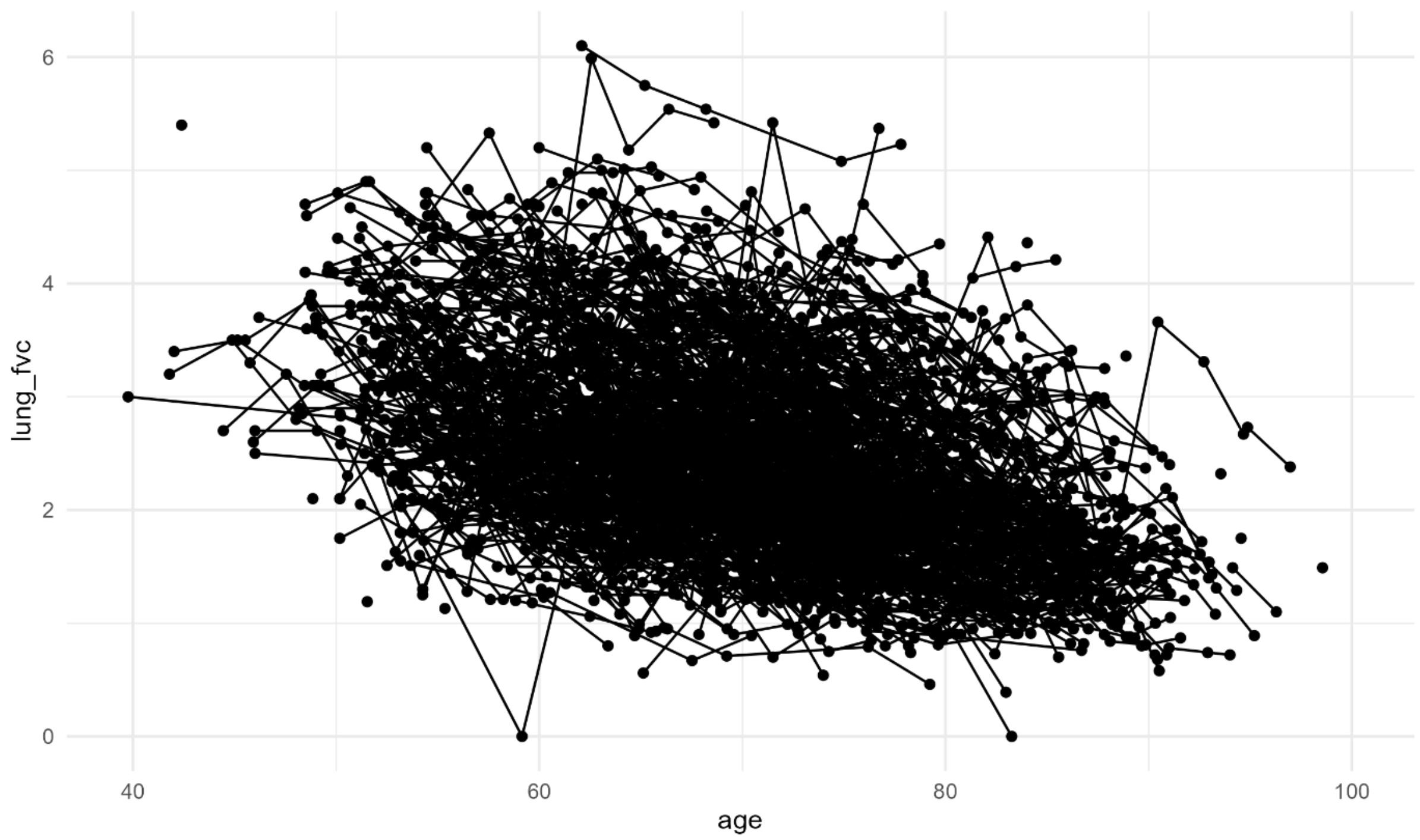

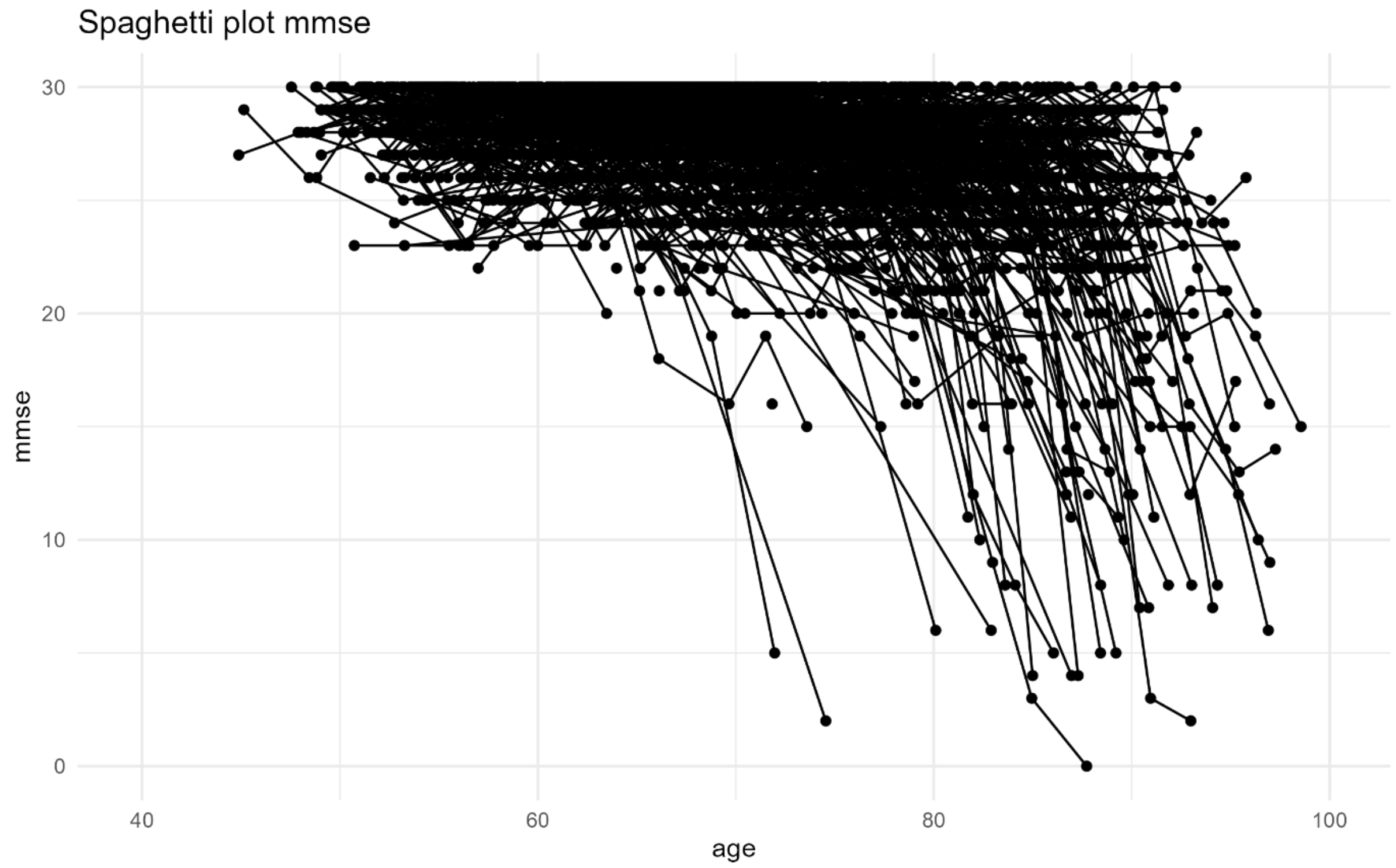

Spaghetti plot mmse
mmse
age
0
10
20
30
40
60
80
100

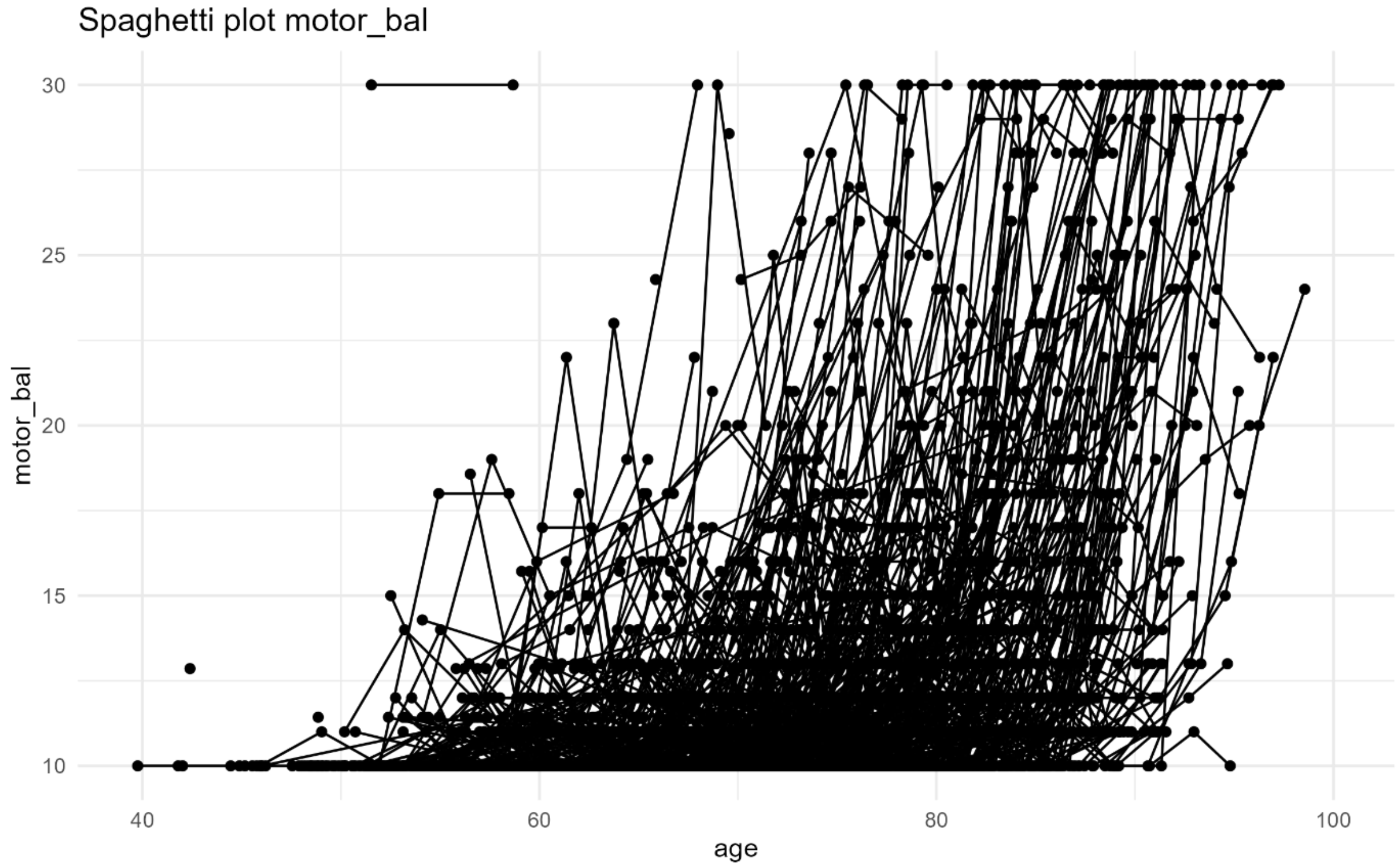

Spaghetti plot motor_bal
motor_bal
age
10
15
20
25
30
40
60
80
100

Spaghetti plot motor_fine

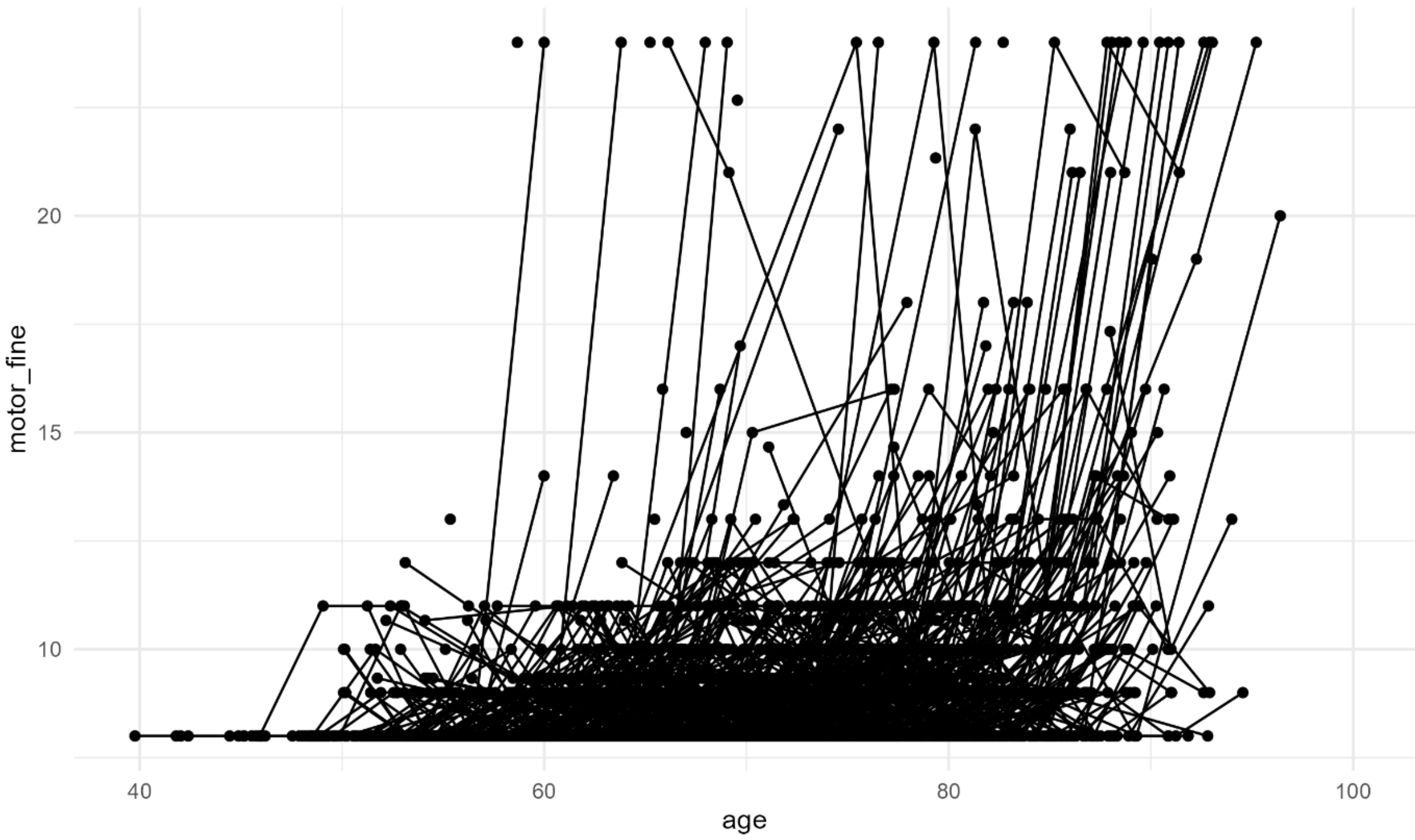

Spaghetti plot motor_flex

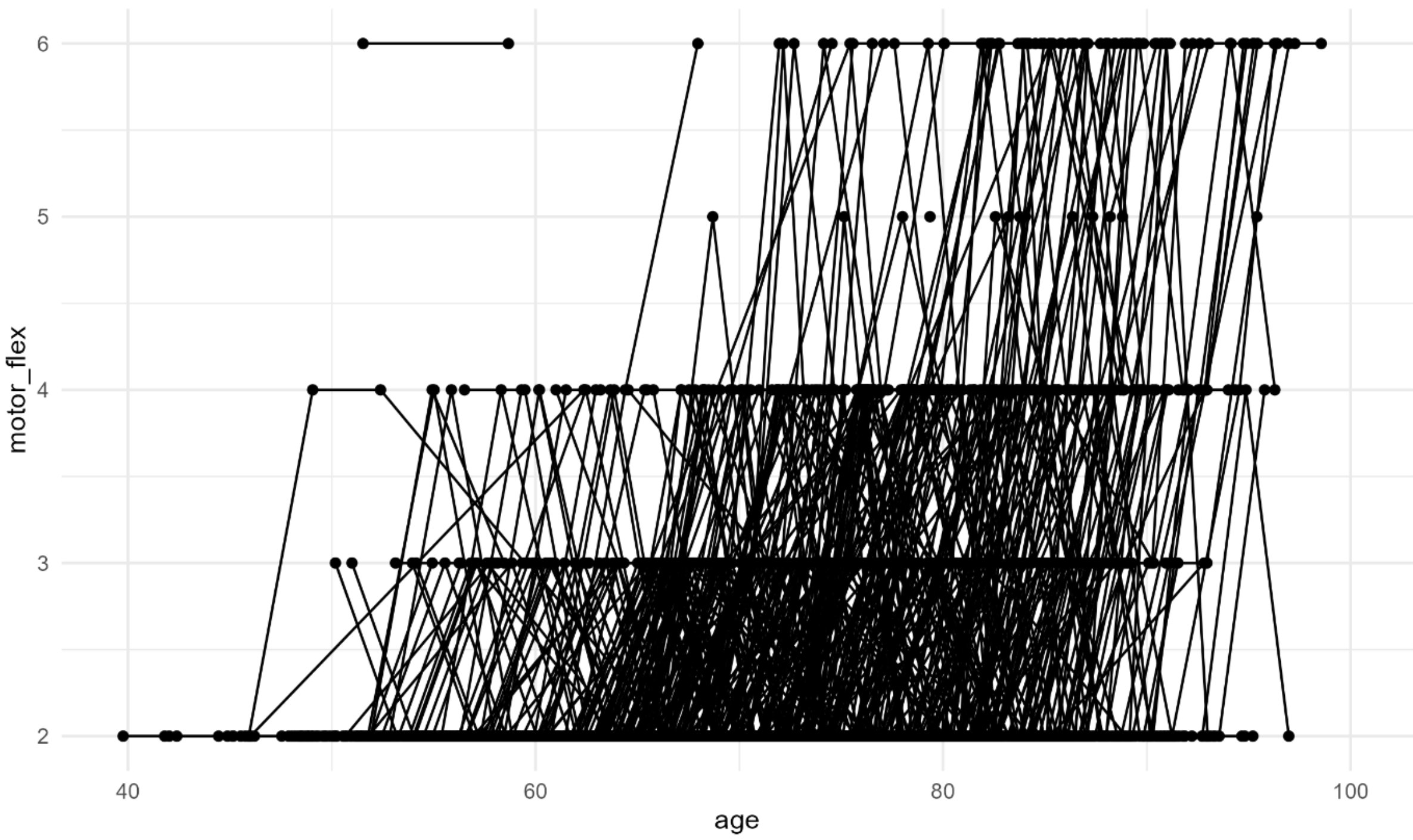

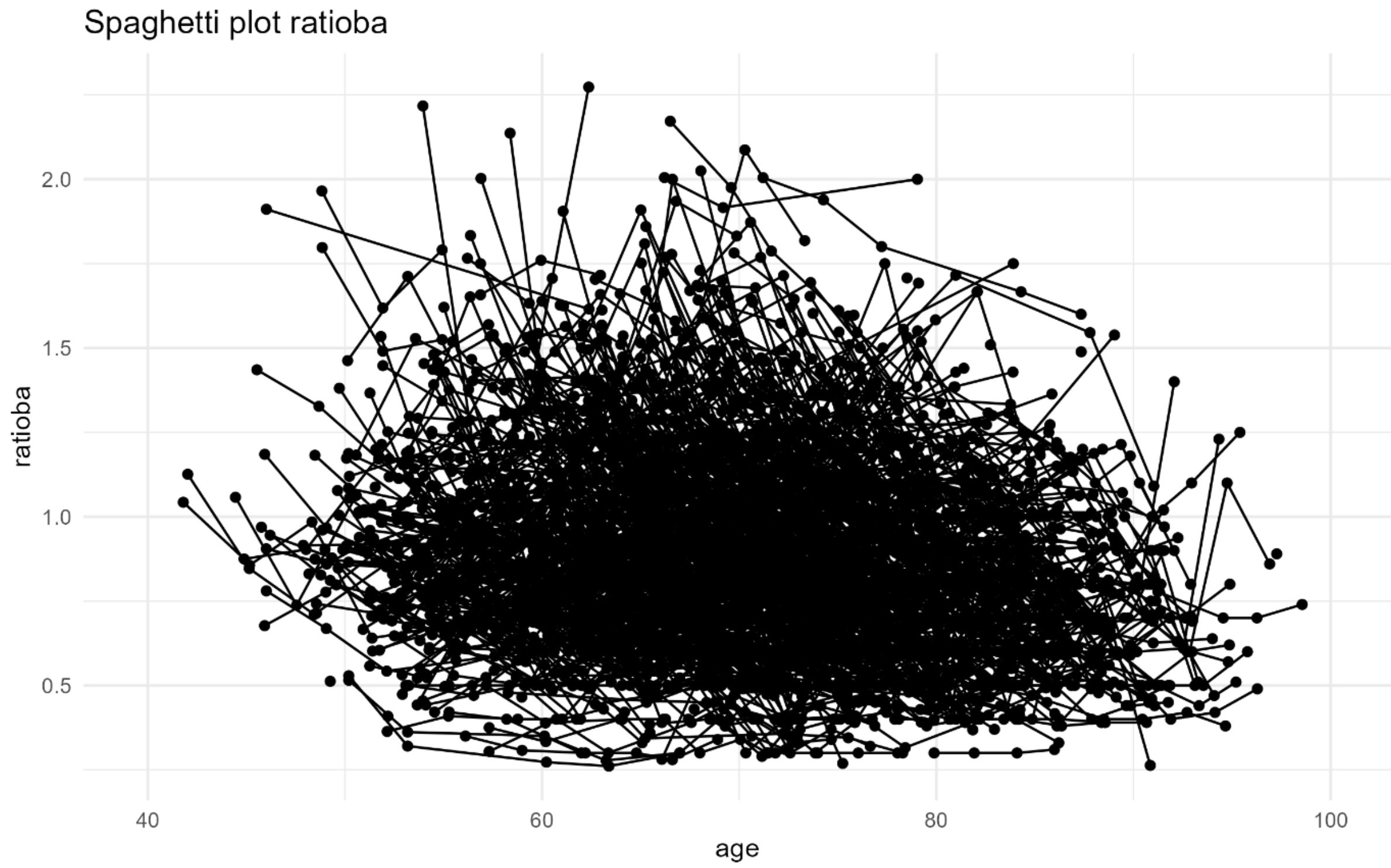
Spaghetti plot ratioba
ratioba
2.0
1.5
1.0
0.5
40
60
80
100
age

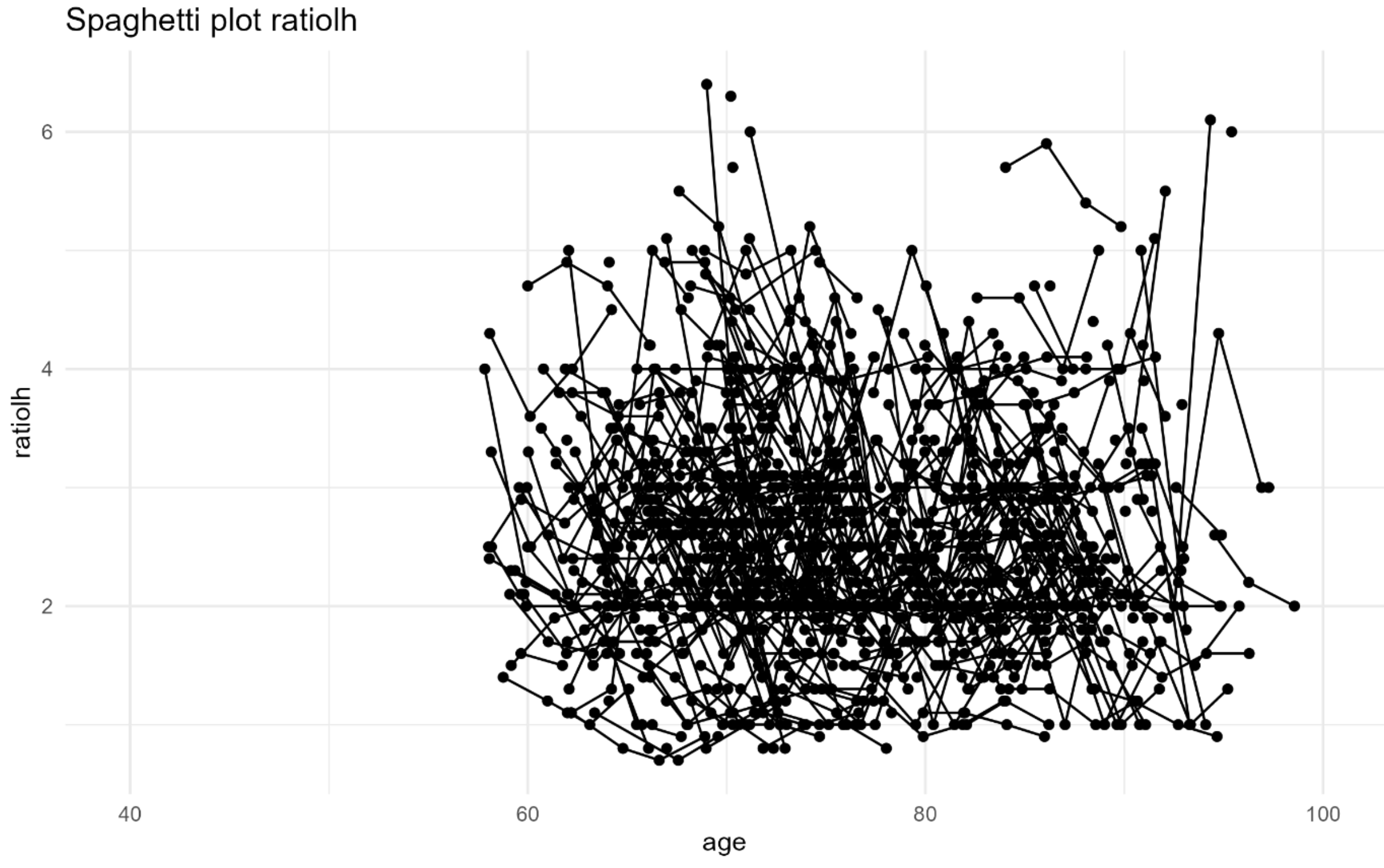
Spaghetti plot ratiolh
ratiolh
6
4
2
40
60
80
100
age

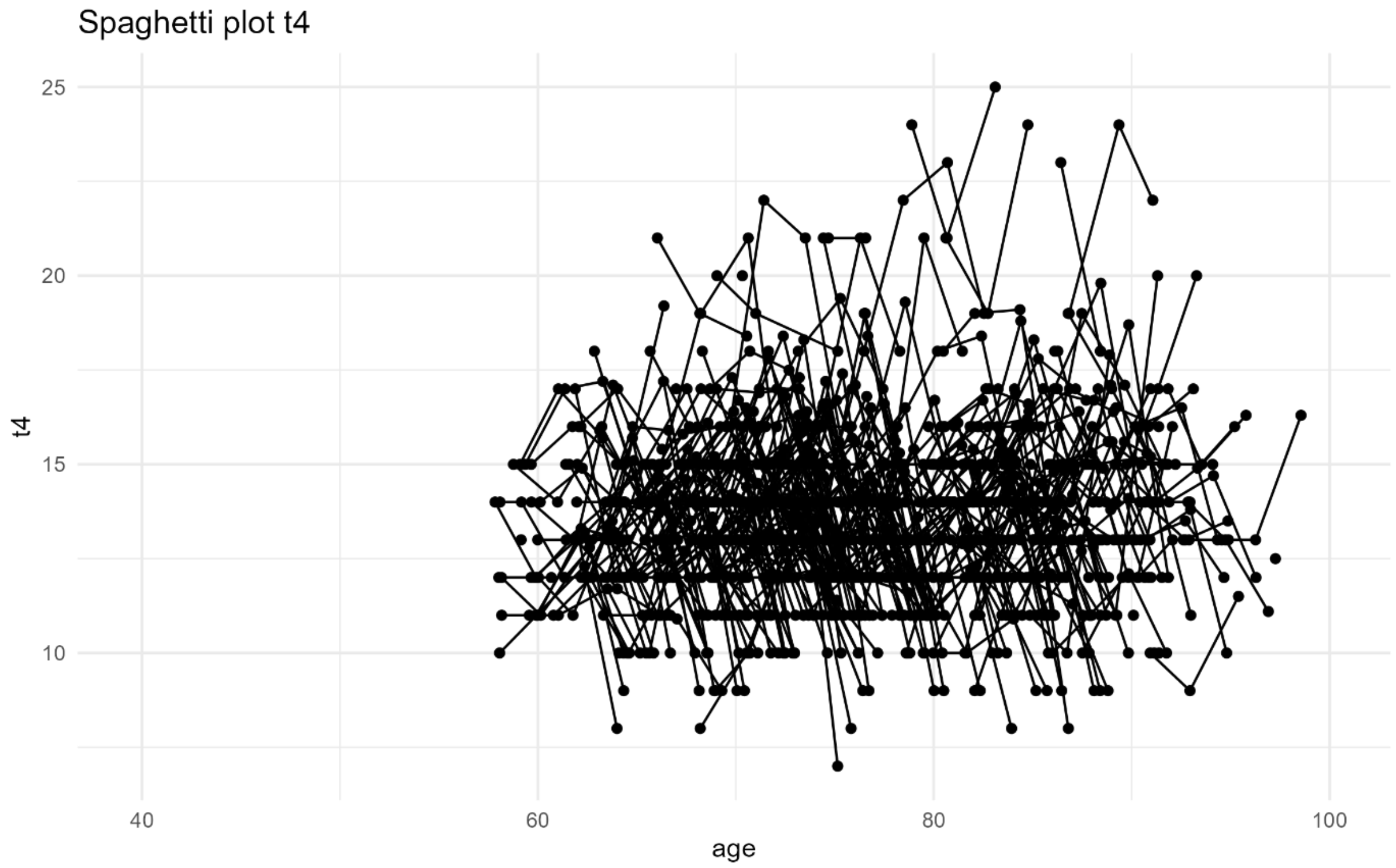
Spaghetti plot t4
25
20
15
10
t4
40
60
80
100
age

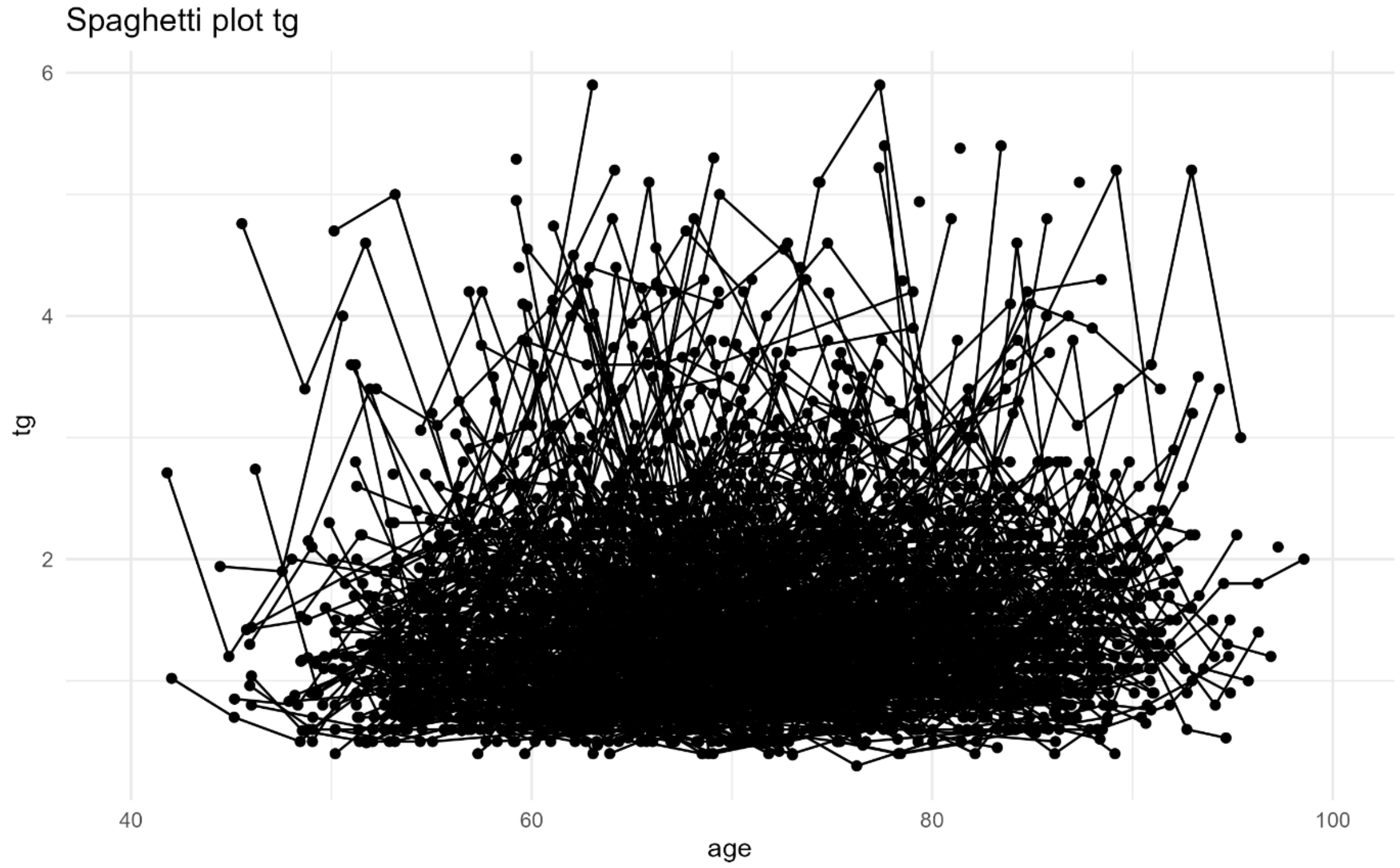
Spaghetti plot tg
6
4
2
tg
40
60
80
100
age

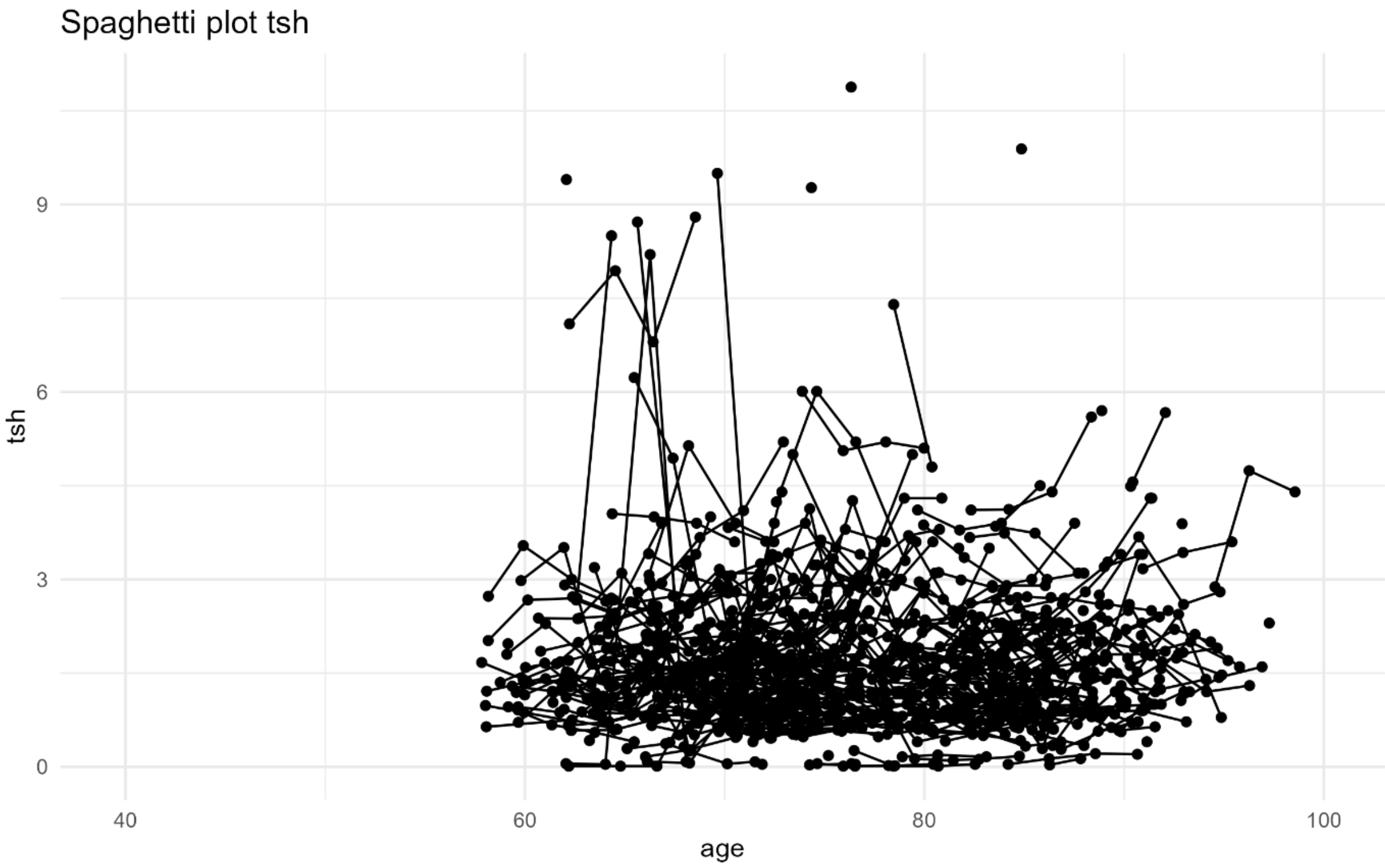

Spaghetti plot tsh
tsh
age
0
3
6
9
40
60
80
100

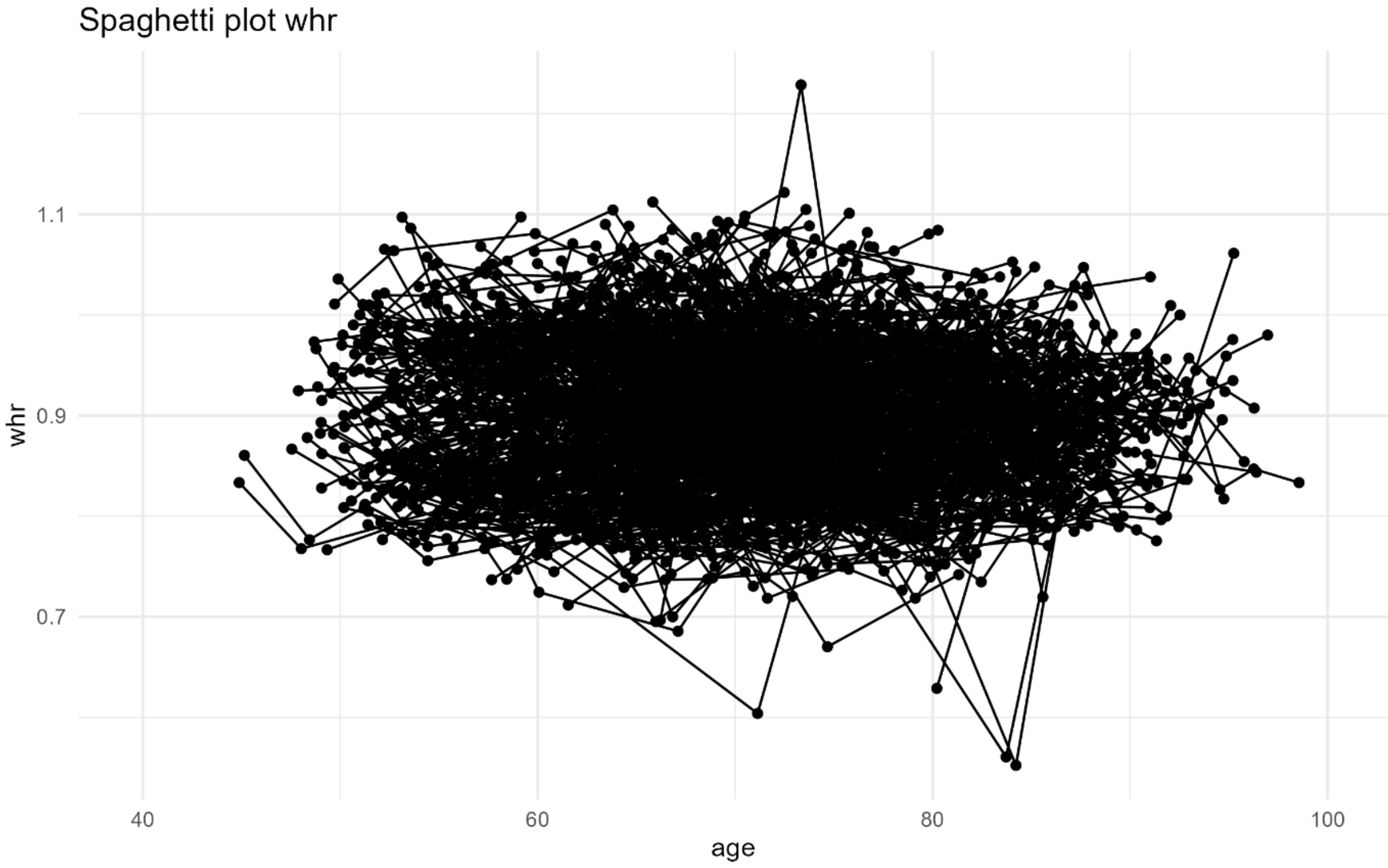

Spaghetti plot whr
whr
age
0.7
0.9
1.1
40
60
80
100

Supplementary Information: Note 2

A generalized approach for estimating the pace of aging

Marije H. Sluiskes, PhD[1,2], Joris Deelen, PhD[1], Hein Putter, PhD[1], Sara Hägg, PhD[2], and Mar Rodriguez-Girondo, PhD*[1]

(m.rodriguez_girondo@lumc.nl)

[1]*Biomedical Data Sciences, Leiden University Medical Center, Leiden, The Netherlands;* [2]*Medical Epidemiology and Biostatistics, Karolinska Institutet, Stockholm, Sweden*

This Note contains plots of the fixed effects of the mixed-effects models fitted on 16 different biomarkers: the 10 biomarkers that were used in the construction of the weighted Pace of Aging, the 4 lipid-related markers that were excluded due to an unexpected relation with age attributed to lipid-altering medication use (cholesterol, ApoA1, ApoB, ratio ApoB/ApoA1) and the 2 markers whose effect on mortality is non-monotone (BMI, triglycerides). See section 2.3 of the main manuscript for the mixed-effects model formulation. See Note 1 for more details on the biomarker definitions.

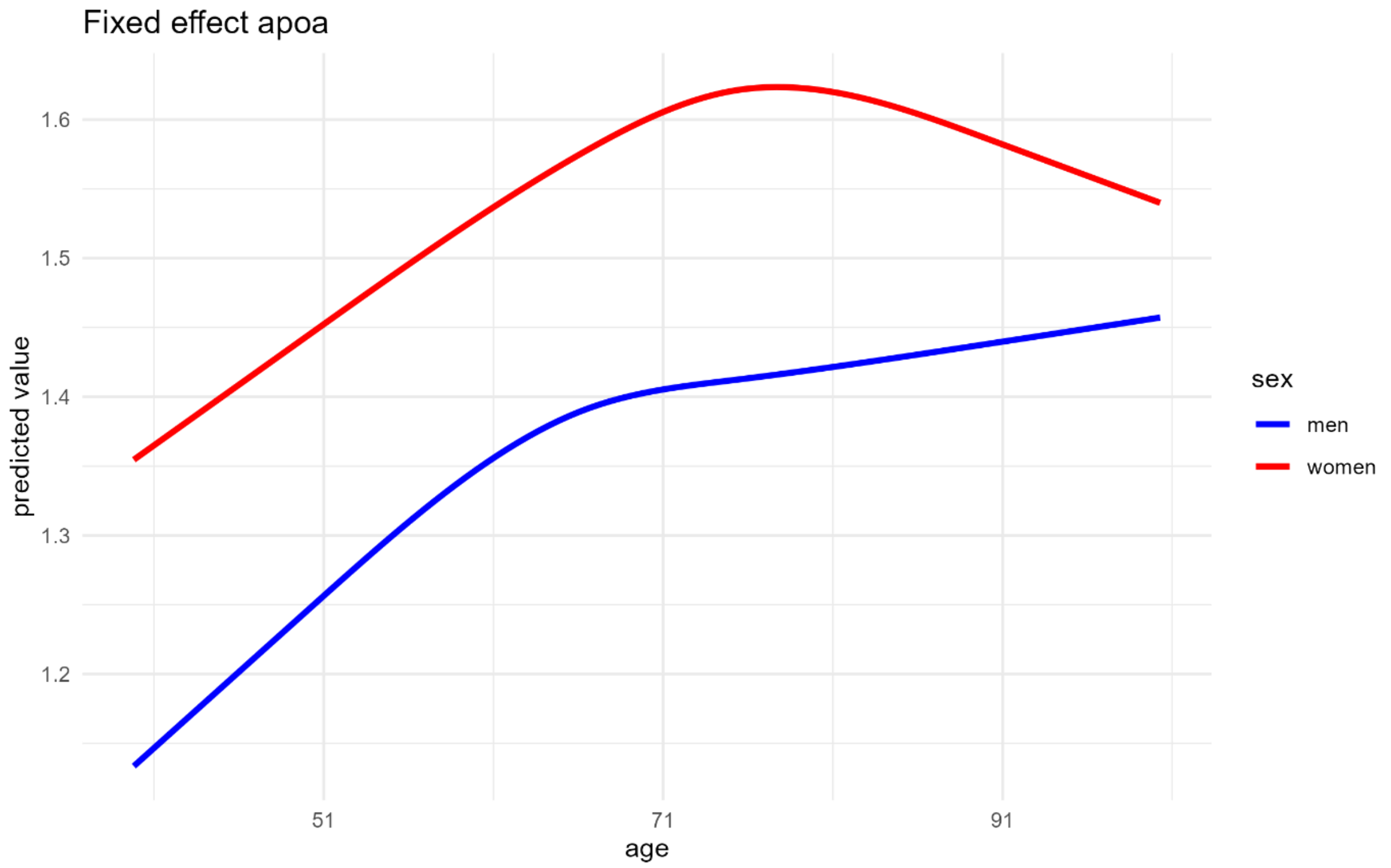
Fixed effect apoa
predicted value
1.6
1.5
1.4
1.3
1.2
51
71
91
age
sex
men
women

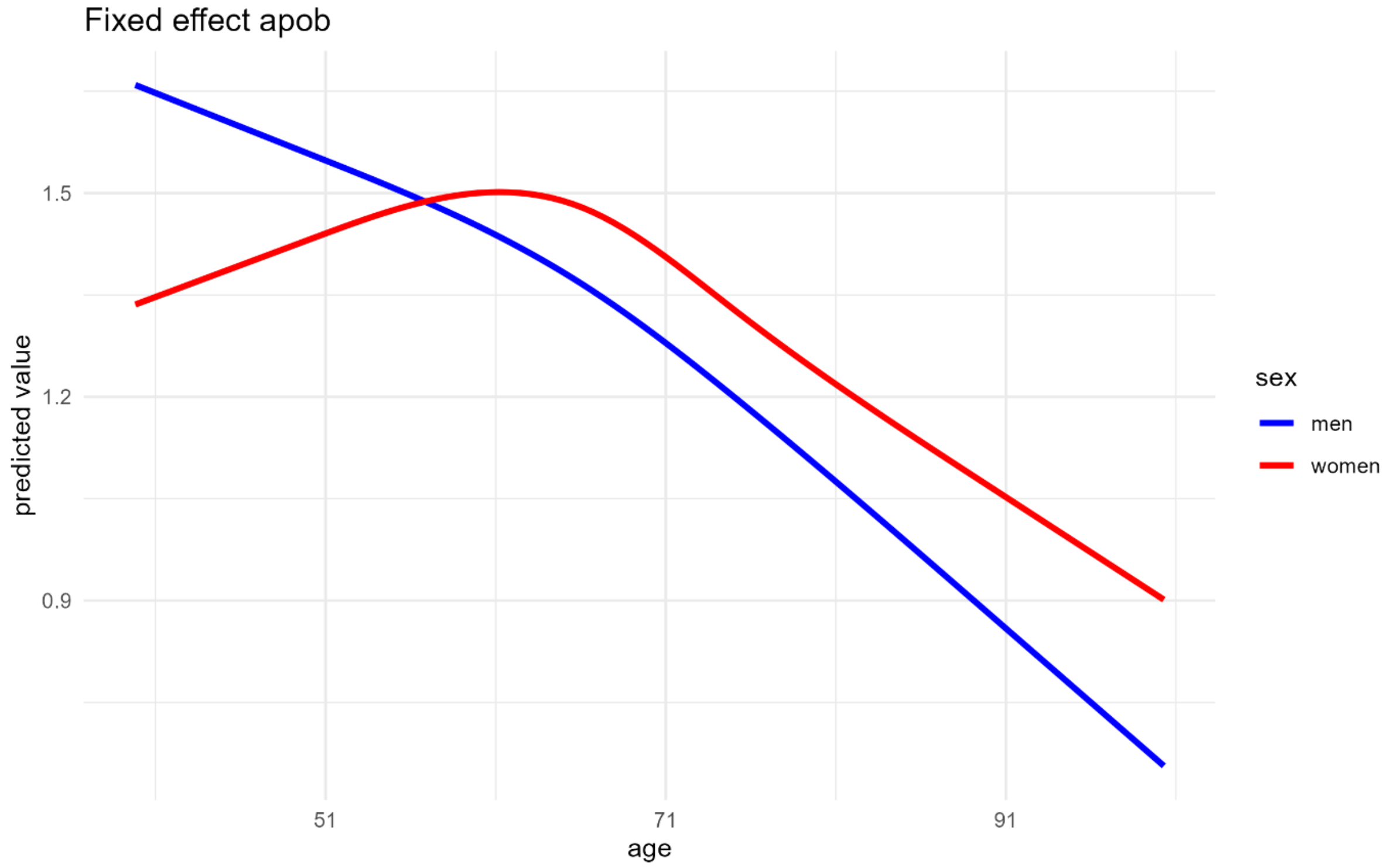
Fixed effect apob
predicted value
1.5
1.2
0.9
51
71
91
age
sex
men
women

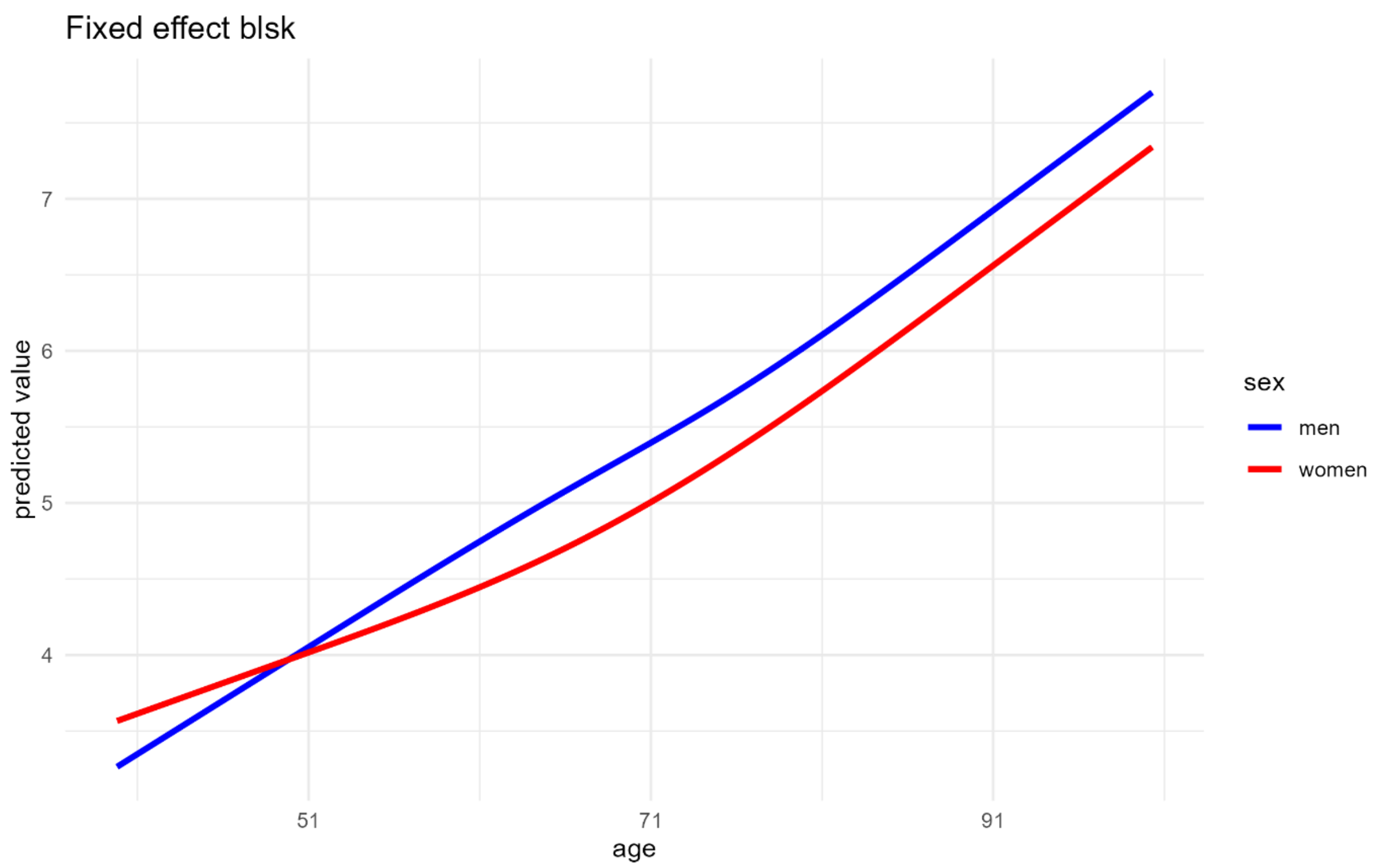
Fixed effect blsk
predicted value
age
4
5
6
7
51
71
91
sex
men
women

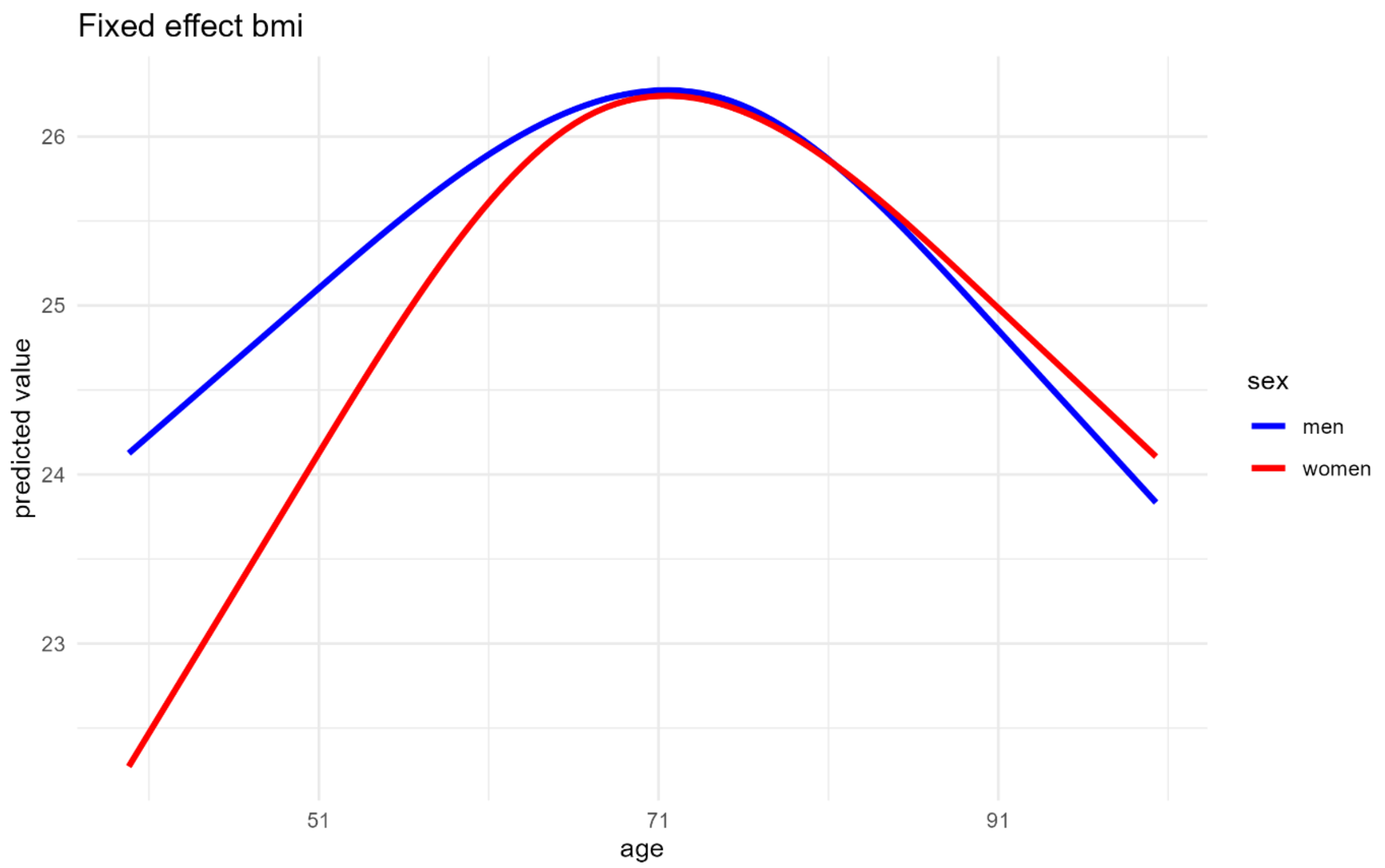
Fixed effect bmi
predicted value
age
23
24
25
26
51
71
91
sex
men
women

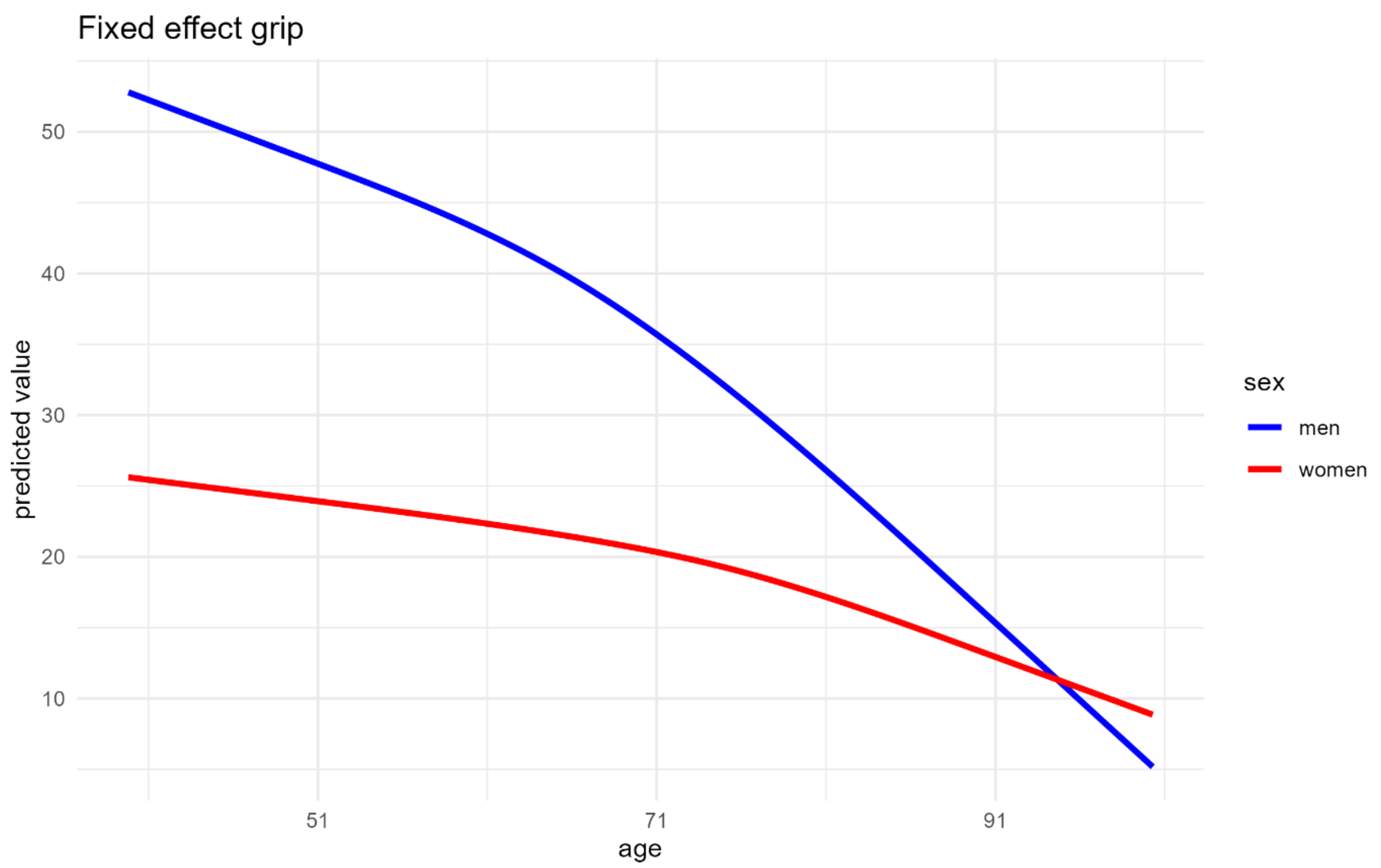
Fixed effect grip
predicted value
50
40
30
20
10
51
71
91
age
sex
men
women

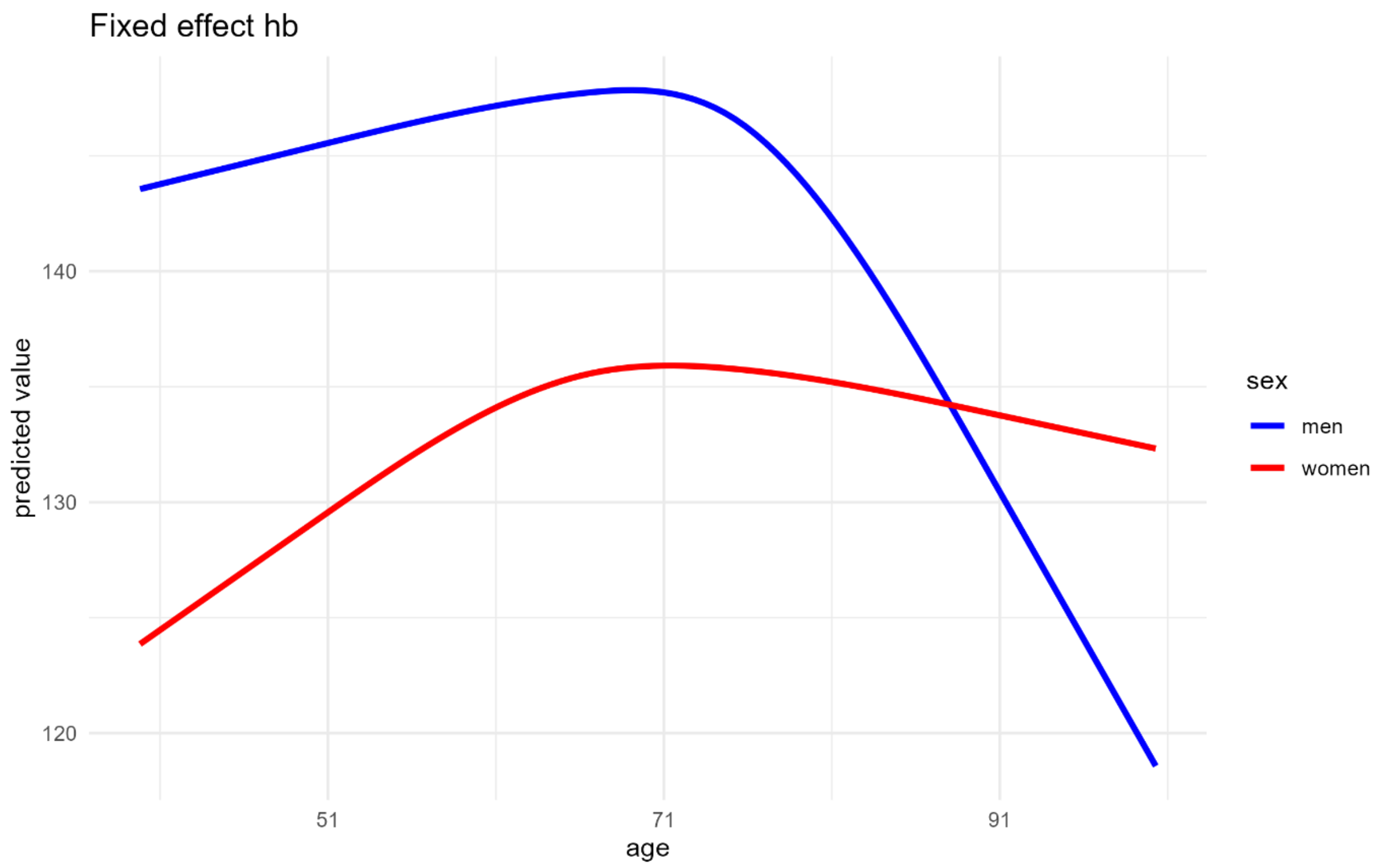
Fixed effect hb
predicted value
140
130
120
51
71
91
age
sex
men
women

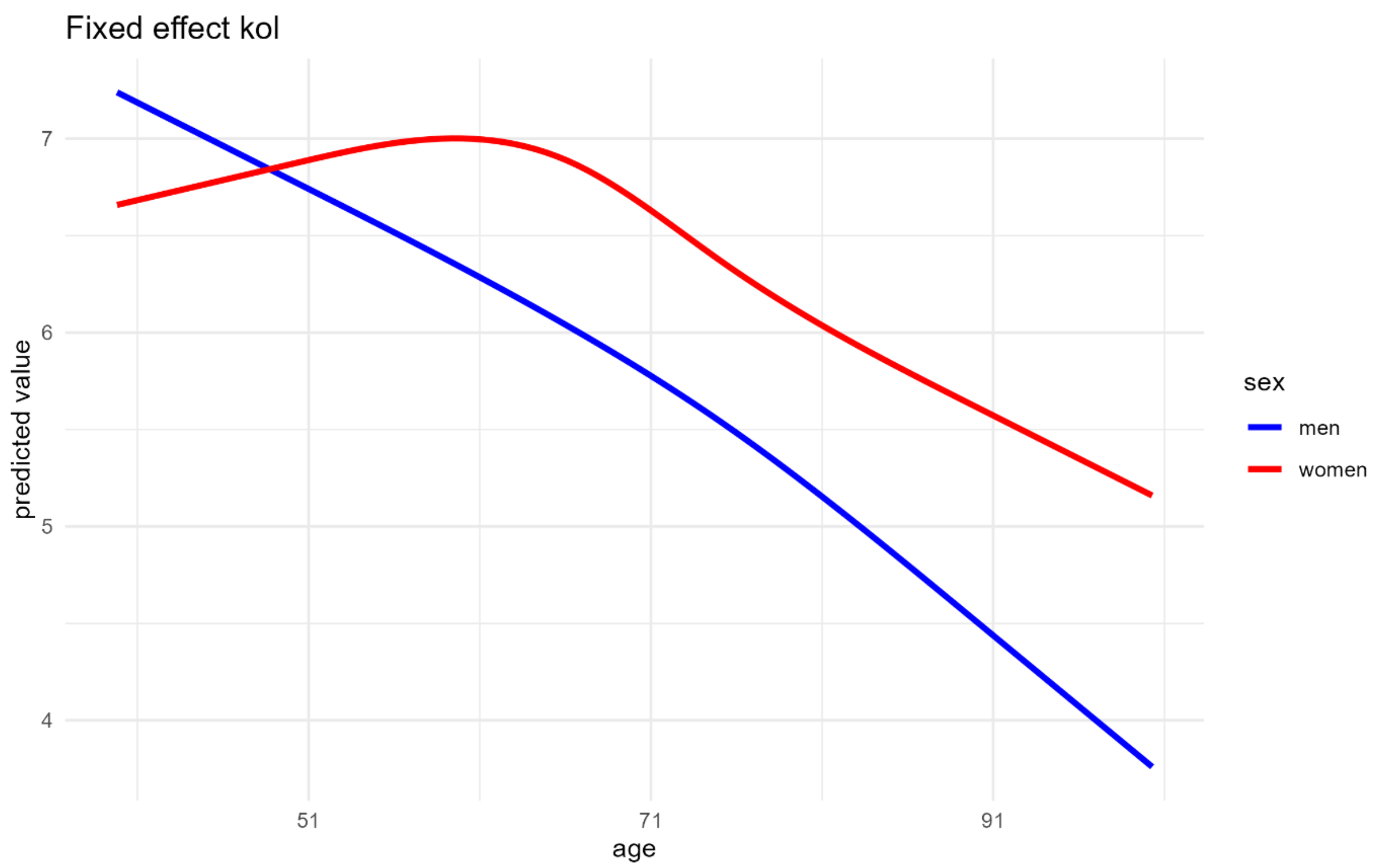
Fixed effect kol
predicted value
7
6
5
4
51
71
91
age
sex
men
women

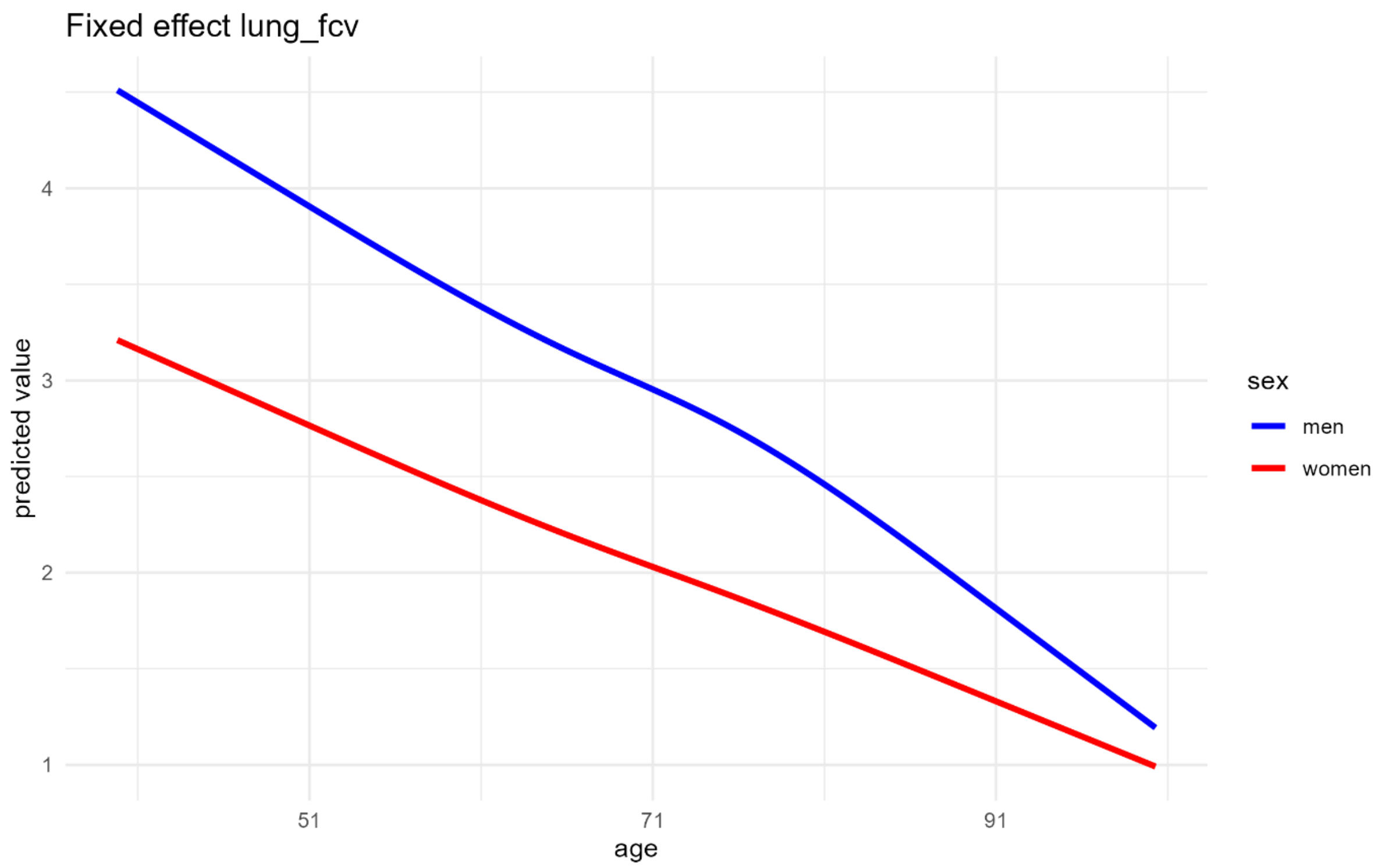
Fixed effect lung_fcv
predicted value
4
3
2
1
51
71
91
age
sex
men
women

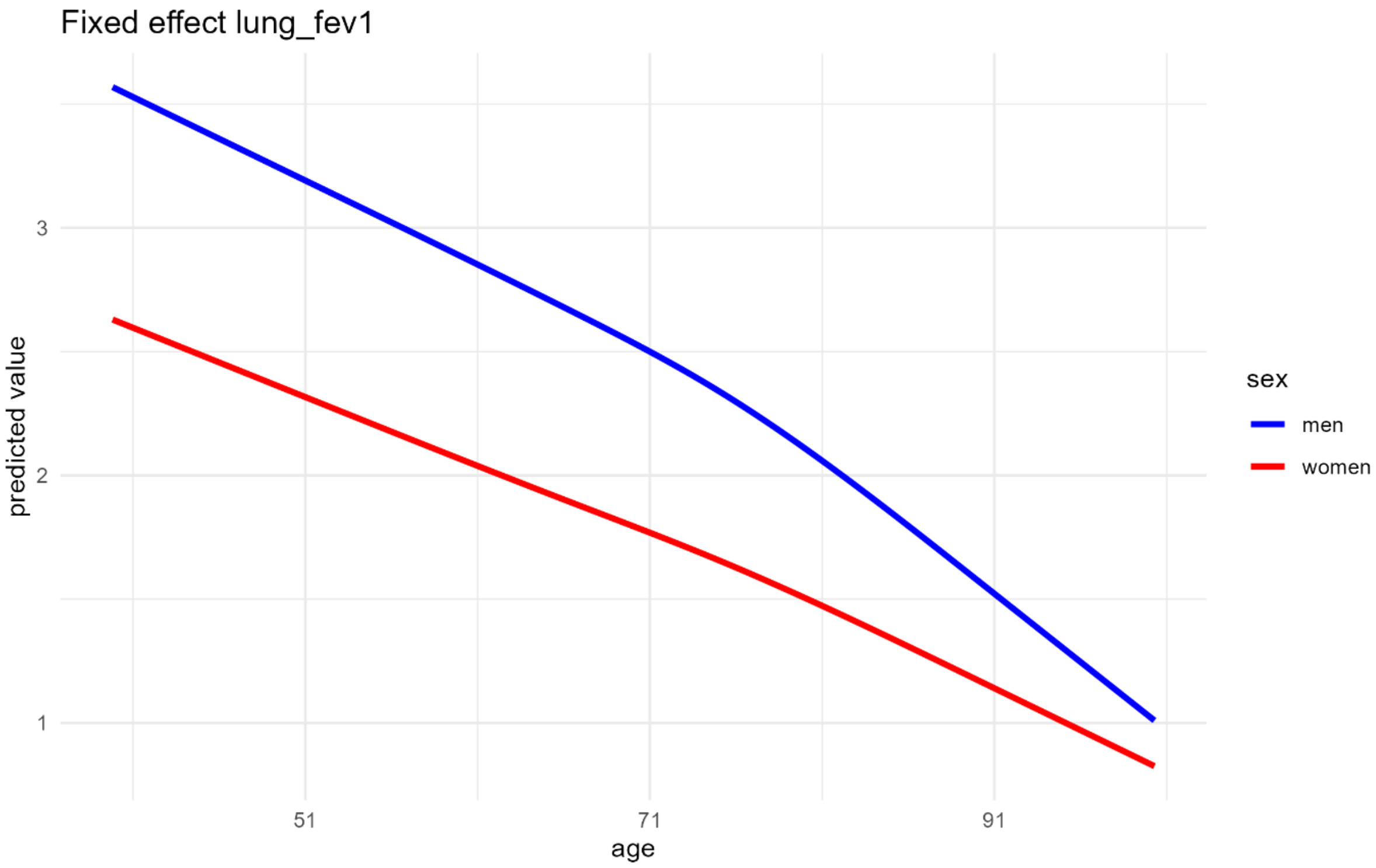
Fixed effect lung_fev1
predicted value
3
2
1
51
71
91
age
sex
men
women

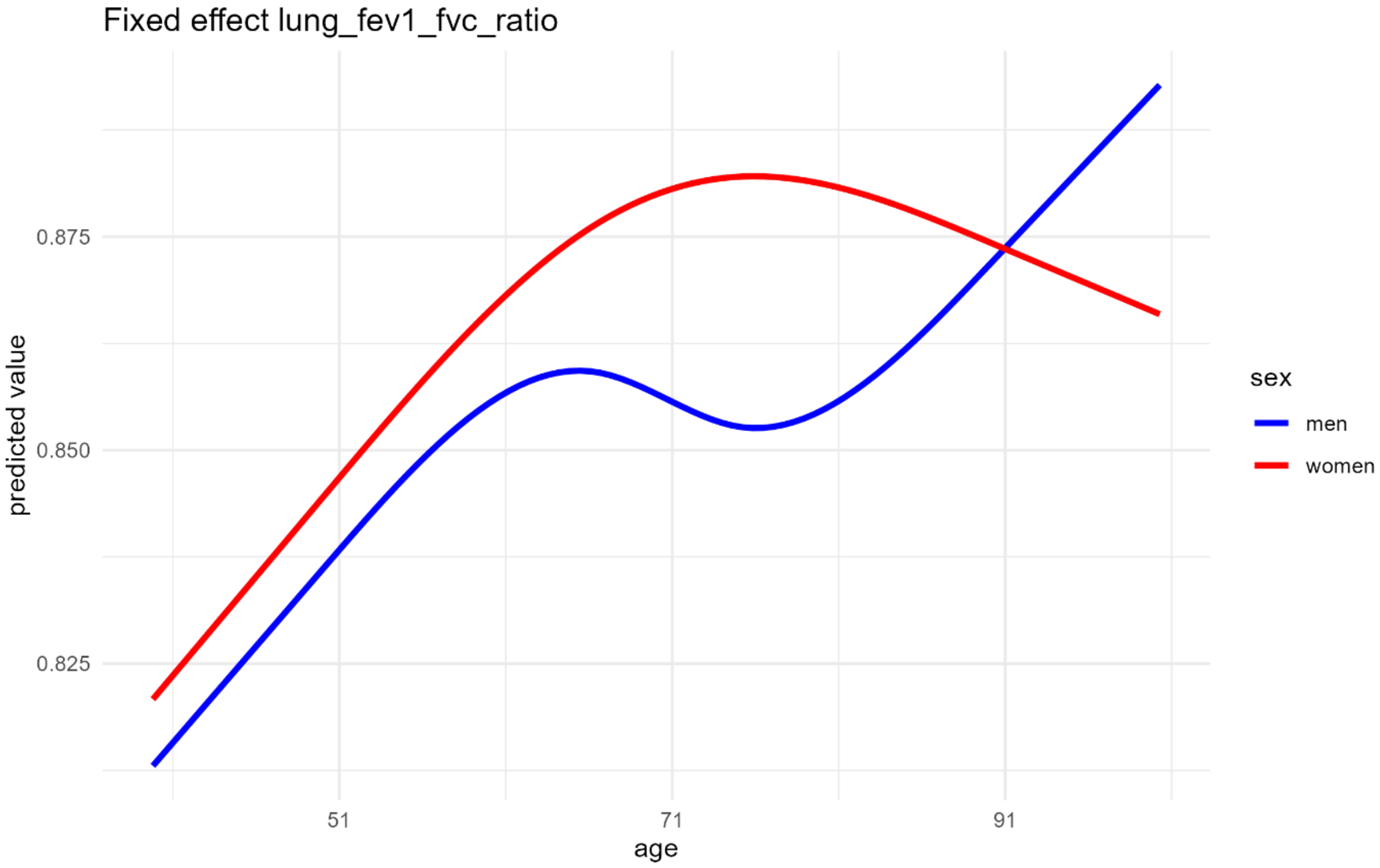
Fixed effect lung_fev1_fvc_ratio
predicted value
0.875
0.850
0.825
51
71
91
age
sex
men
women

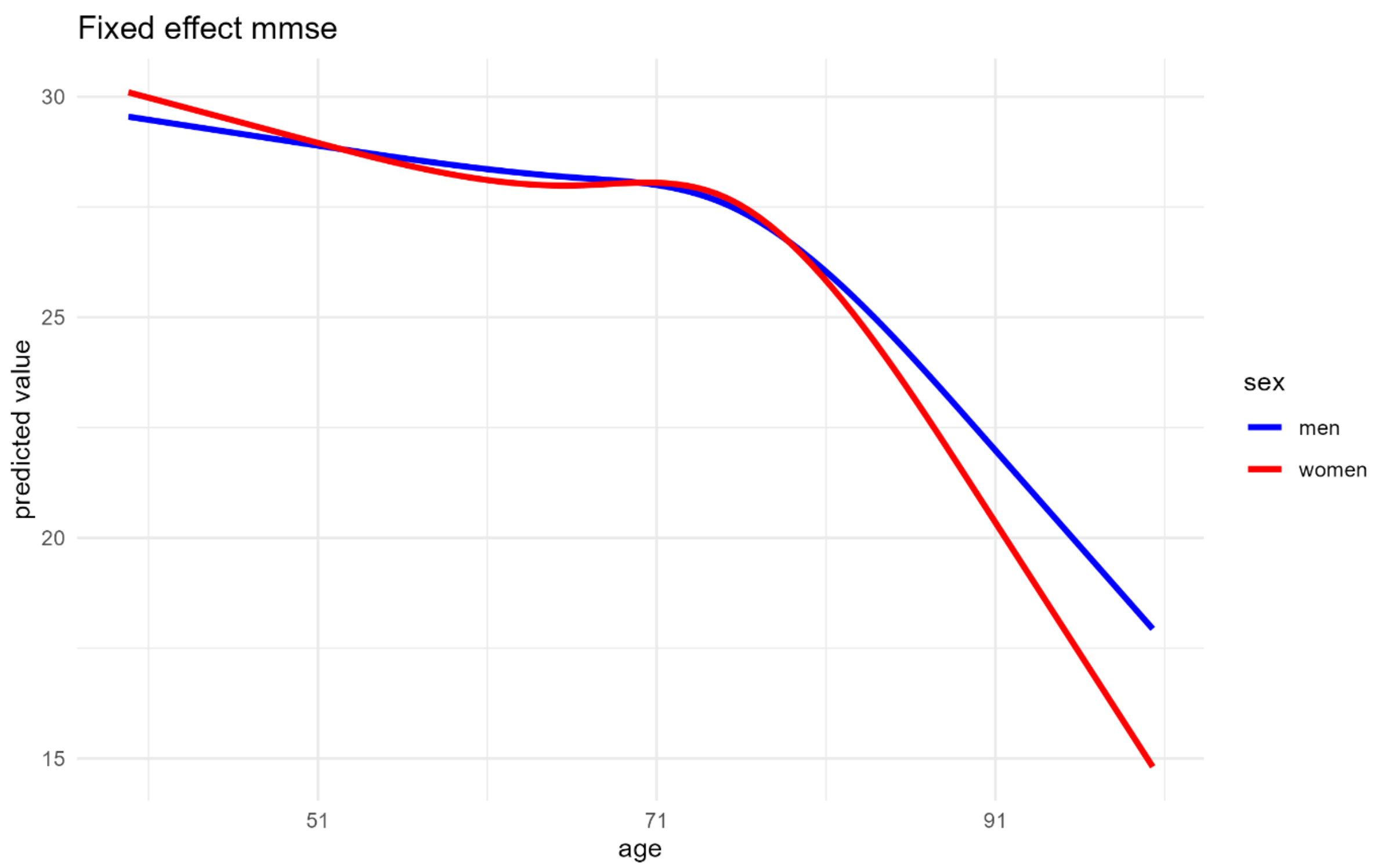
Fixed effect mmse
predicted value
30
25
20
15
51
71
91
age
sex
men
women

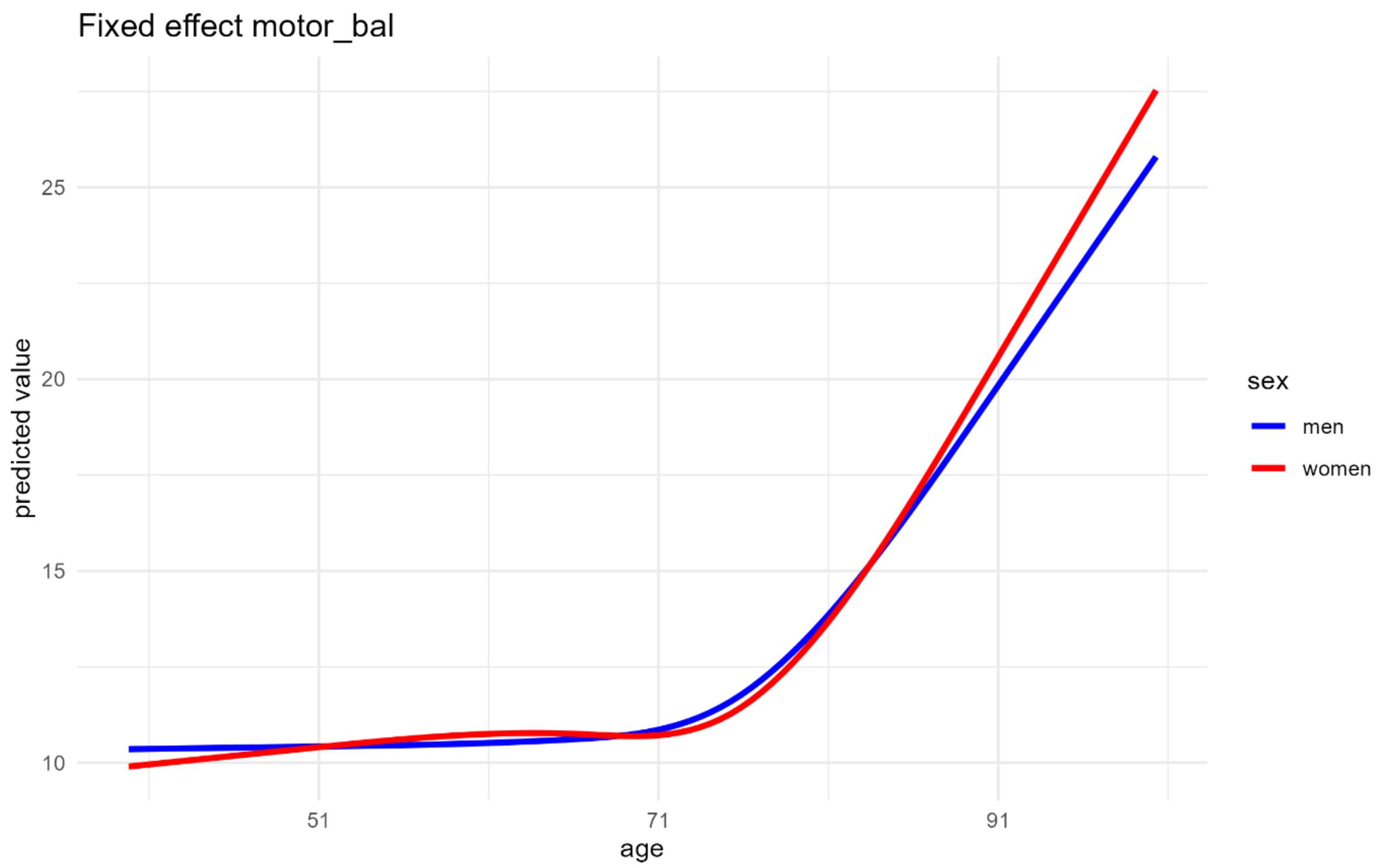
Fixed effect motor_bal
predicted value
25
20
15
10
51
71
91
age
sex
men
women

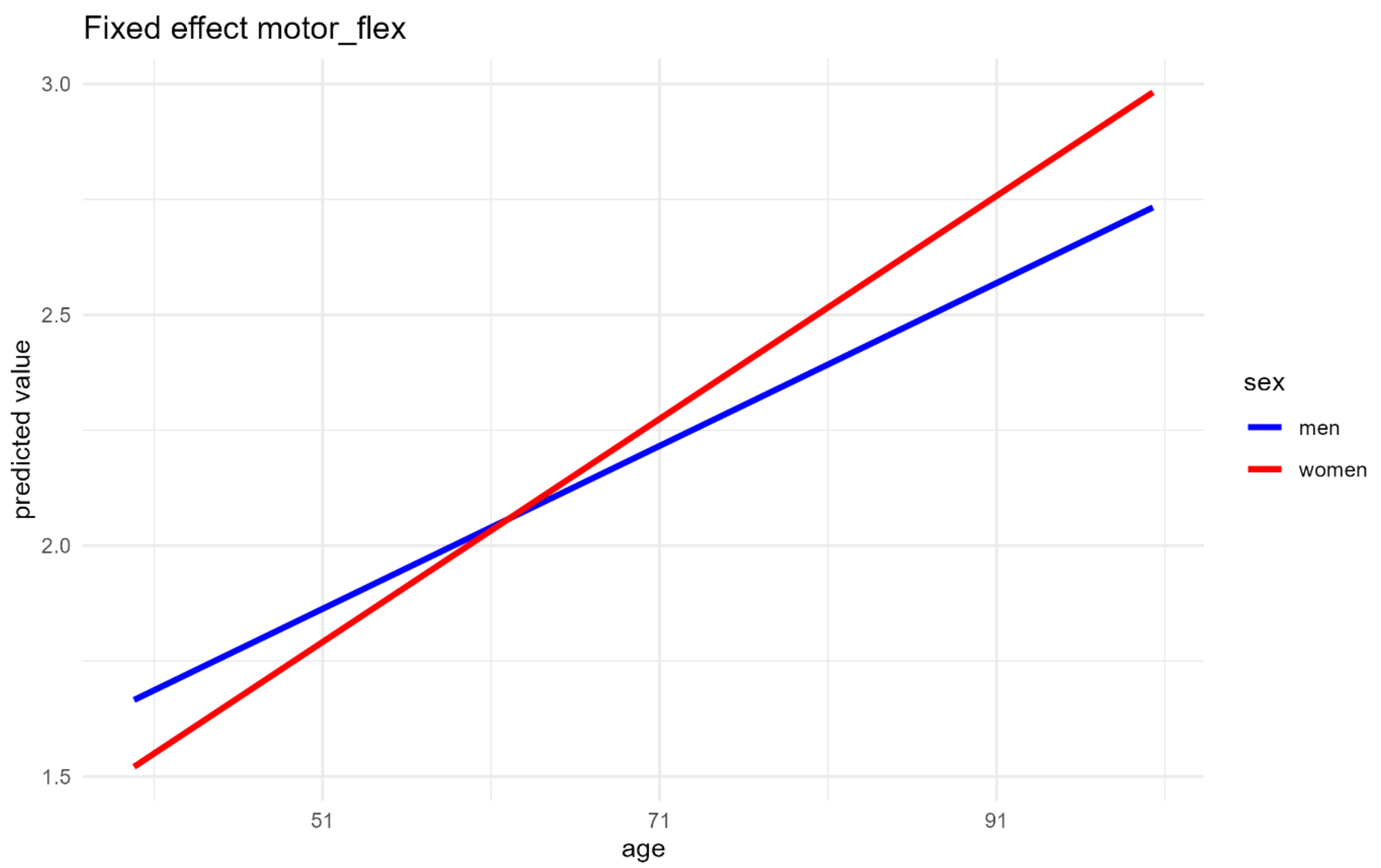
Fixed effect motor_flex
predicted value
3.0
2.5
2.0
1.5
51
71
91
age
sex
men
women

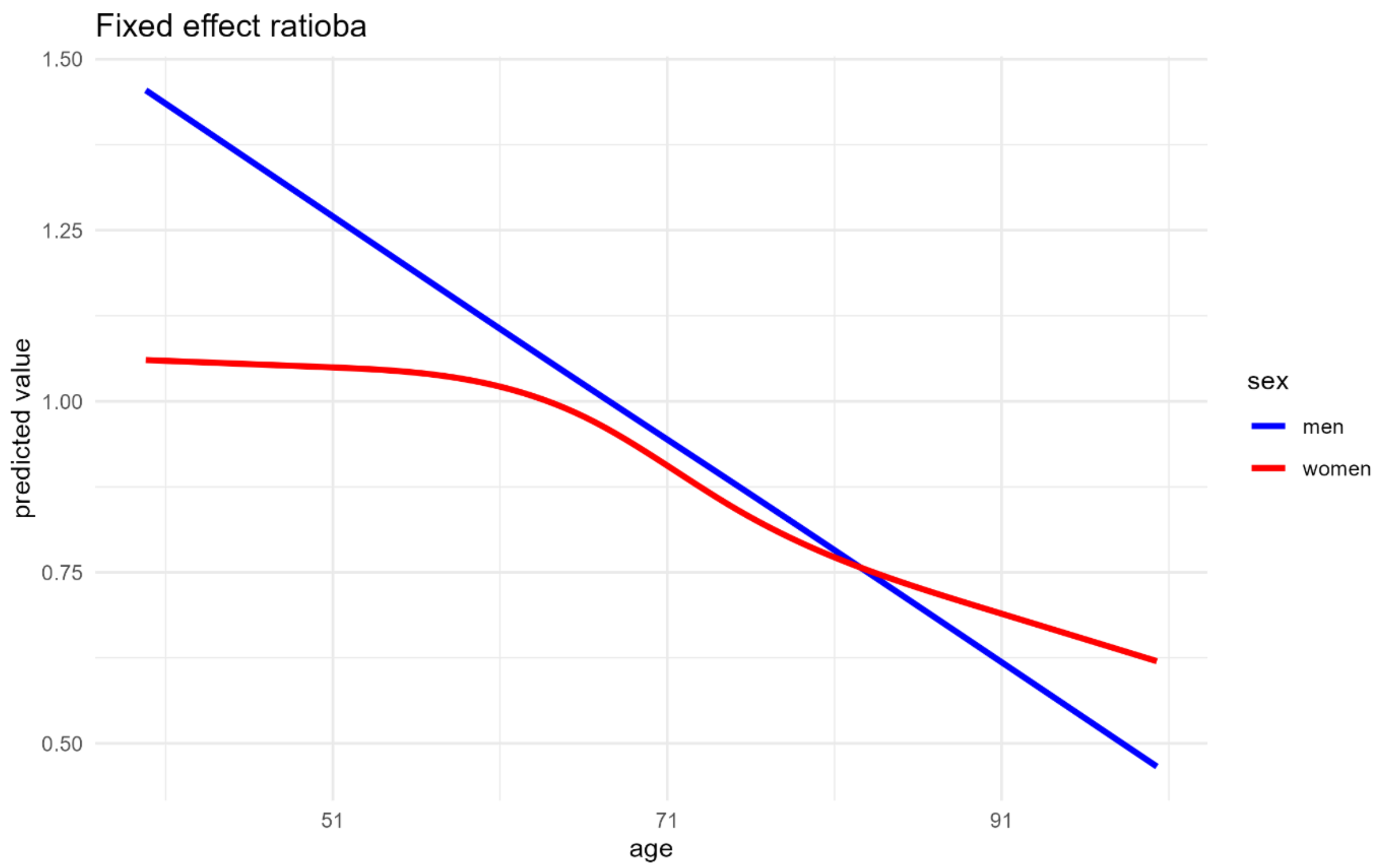
Fixed effect ratioba
predicted value
1.50
1.25
1.00
0.75
0.50
51
71
91
age
sex
men
women

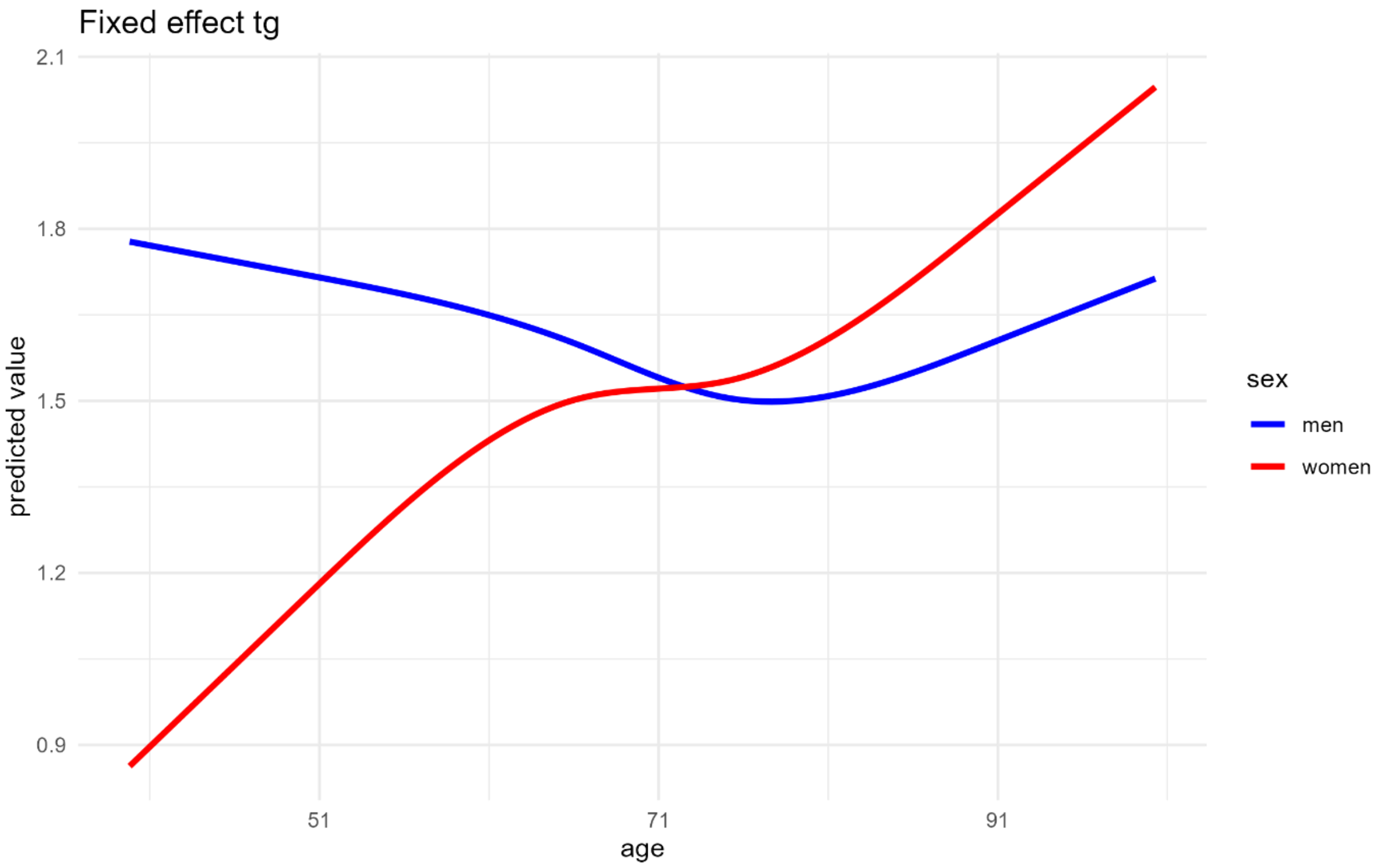

Fixed effect tg
2.1
1.8
1.5
1.2
0.9
predicted value
51
71
91
age
sex
men
women

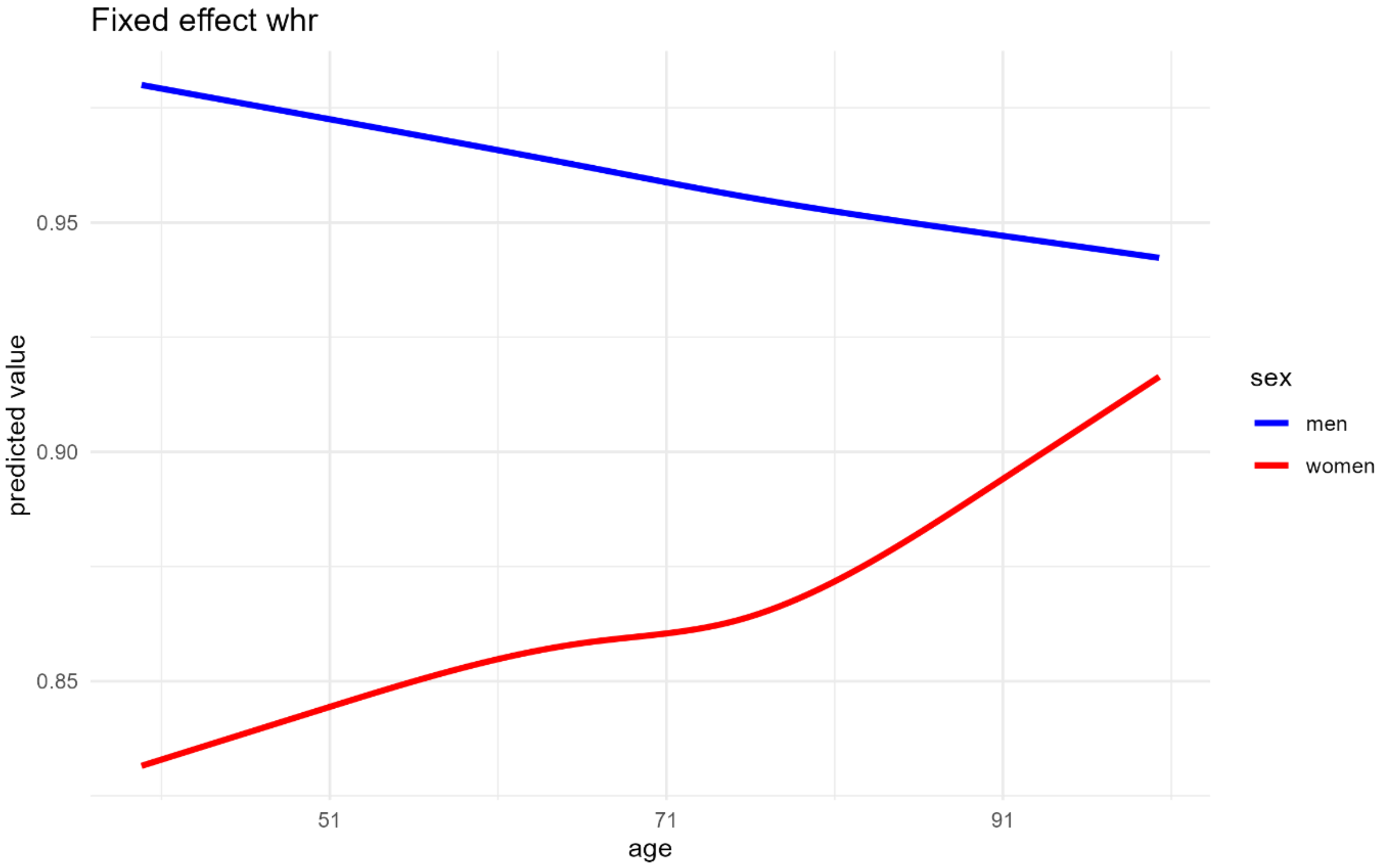

Fixed effect whr
0.95
0.90
0.85
predicted value
51
71
91
age
sex
men
women

Supplementary Information: Note 3

A generalized approach for estimating the pace of aging

Marije H. Sluiskes, PhD[1,2], Joris Deelen, PhD[1], Hein Putter, PhD[1], Sara Hägg, PhD[2], and Mar Rodriguez-Girondo, PhD*[1] (m.rodriguez_girondo@lumc.nl)

[1]*Biomedical Data Sciences, Leiden University Medical Center, Leiden, The Netherlands;* [2]*Medical Epidemiology and Biostatistics, Karolinska Institutet, Stockholm, Sweden*

This Note contains detailed information on the construction of the wPoA* the weighted pace of Aging based on external weights. Moreover, it contains the weights for both wPoA and wPoA* (Table S2).

To construct wPoA*, we proceeded as follows. We identified relevant estimates from a targeted (non-systematic) literature review reporting associations between baseline biomarkers and mortality, primarily from Cox proportional hazards models. Where possible, we used estimates from large population-based cohorts such as UK Biobank and NHANES; however, this was not always feasible. Specifically, for clinical biomarkers such as lung function and blood sugar, we relied on UK Biobank (Rydell et al., 2024) and a large study including data from 97 studies (Seshasai et al. (2011); covering NHANES and other large European cohorts), respectively, with broadly

comparable definitions to those used in SATSA. For grip strength, we relied on a study based on UK Biobank (Kim et al., 2017). For waist-to-hip ratio, we used a large meta-analysis on the association between adiposity-related measures and all-cause mortality in older adults based on multiple aging cohorts (Alharbi et al., 2021). Similarly, for MMSE, we relied on a large meta-analysis based on several aging cohorts (Rostamian et al., 2022). Comparable studies based on NHANES/UKB or large meta-analyses were not available for functional balance and flexibility measures, as published evidence linking these measures to mortality remains scarce. For balance and flexibility, we therefore used the largest and most recent available studies with comparable metrics from Korea (Kim et al., 2023) and Brazil (Gil S. Araujo et al., 2024), respectively. Importantly, SATSA was not included in any of these external sources to avoid circularity and ensure that the weights were fully independent from the dataset used for evaluation. Whenever possible, we selected adjusted hazard ratios to better reflect multivariable relationships consistent with our framework. When hazard ratios were reported for categorized biomarkers, we transformed them to approximate continuous effects on a comparable scale.

To align these estimates with our Gompertz accelerated failure time (AFT) framework, hazard ratios were converted into acceleration factors. This transformation is exact under a Weibull model, as it is the only standard survival model that satisfies both

proportional hazards and accelerated failure time formulations. Specifically, acceleration factors (AF) were obtained from hazard ratios (HR) via $AF = HR-1/k$, where k is the Weibull shape parameter. In this parametrization, larger AFs correspond to faster aging. We assumed a shape parameter of $k=4$, reflecting the increasing hazard with age and consistent with empirical studies of adult mortality, where Weibull models with shape parameters around this value provide a good fit (Moser et al., 2015).

Because FEV1, FVC, and FEV1/FVC are highly correlated, we included only one of these measures, FVC, in the construction of wPoA*. This also illustrates the potential advantage of a data-driven approach for handling correlated biomarkers since finding appropriate published literature in that case is difficult. Because of lack of appropriate literature, we also excluded Hemoglobin from the computation of wPoA*. The list of selected biomarkers, along with the corresponding published hazard ratios and derived acceleration factors, is provided in Supplementary Table S1 below and in the Supplementary Note 4 in the revised version of the manuscript.

Using this same reduced set of biomarkers, we also computed an unweighted version, uPoA*, by summing the random slopes, allowing us to reconstruct Table 2. Overall, wPoA* shows slightly larger hazard ratios than its unweighted counterpart, illustrating the added value of weighting. However, the

differences are modest; accordingly, we have moderated our claims regarding the superiority of the weighted approach in the specific SATSA setting and instead emphasize the conceptual contribution of the proposed new framework.

Supplementary Table S1. Summary of SATSA-derived acceleration factors (AF), external hazard ratios (HR), and corresponding externally derived AFs (wPoA* weights). External HRs were obtained from published studies and transformed to be comparable across biomarkers. To ensure consistency with the longitudinal modeling framework, all effects were oriented such that a higher (more positive) slope corresponds to higher mortality risk. For biomarkers that decline with age (e.g., grip strength, MMSE, lung function), reported HRs were inverted so that positive slope deviations (slower decline or improvement) correspond to lower risk. External AFs were derived from HRs assuming a Weibull baseline hazard, using AF = HR−1/k with shape parameter k = 4. Biomarker-specific transformations included: (i) motor flexibility, where sex-specific HRs from grouped comparisons were first log-transformed and sex-specific values averaged, then converted to a per-unit effect by dividing by the approximate difference between groups, and finally rescaled to the SATSA metric using the ratio of standard deviations, yielding an approximate HR per SATSA unit; (ii) motor balance, where a binary HR was converted to a per-unit effect using the observed standard

deviation; and (iii) lung function (FVC), where HRs based on categorical thresholds were transformed to continuous effects using the difference between reported groups and unit conversions to liters. Due to high correlation among lung function measures, only FVC was included. Hemoglobin was excluded due to lack of suitable external estimates. Based on the same subset of biomarkers, an unweighted version (uPoA*) was also constructed by summing the corresponding random slopes.

Supplementary Table S2. Summary of SATSA-derived acceleration factors (AF, with standard error (S.E.) in brackets), external hazard ratios (External HR), and corresponding externally derived AFs (External AF; wPoA weights). External HRs were obtained from published studies and transformed to be comparable across biomarkers. To ensure consistency with the longitudinal modeling framework, all effects were oriented such that a higher (more positive) slope corresponds to higher mortality risk. For biomarkers that decline with age (e.g., grip strength, MMSE, lung function), reported HRs were inverted so that positive slope deviations (slower decline or improvement) correspond to lower risk. External AFs were derived from HRs assuming a Weibull baseline hazard, using $AF = HR^{-1/k}$ with shape parameter $k = 4$. Biomarker-specific transformations included: (i) motor flexibility, where sex-specific HRs from grouped comparisons were first log-transformed and sex-specific values averaged, then converted to a per-unit effect by dividing by the approximate difference between groups, and finally rescaled to the SATSA metric using the ratio of standard deviations, yielding an approximate HR per SATSA unit; (ii) motor balance, where a binary HR was converted to a per-unit effect using the observed standard deviation; and (iii) lung function (FVC), where HRs based on categorical thresholds were transformed to continuous effects using the difference between reported groups and unit conversions to liters. Due to high correlation among lung function measures, only FVC was included. Hemoglobin was excluded due to lack of suitable external estimates. Based on the same subset of biomarkers, an unweighted version (uPoA*) was also constructed by summing the corresponding random slopes.*

**Biomarker**	**SATSA AF**	**External AF**	**External HR**	**Source**
Hemoglobin	0.993 (0.003)	-	-	
Blood sugar	1.018 (0.017)	1.02	1.10	N Engl J Med 2011;364:829-41
Waisthip ratio	0.658 (0.764)	0.89	0.63	Age and Ageing 2021; 50: 697–704
grip	0.983 (0.006)	0.99	0.98	Am J Clin Nutr. 2017; 106:773–782
mmse	0.989 (0.004)	0.98	0.91	PLoS One. 2022;17:e0261826
Flexibility	1.008 (0.019)	1.08	1.35	Scand J Med Sci Sports 2024, 34: e14708.
Balance	1.007 (0.004)	1.01	1.056	Eur J Med Res 2023; 28:455
FEV1	1.322 (0.161)	-	-	
FVC	0.675 (0.122)	0.83	0.48	Respir Med 2024; 227:107614
FEV1/FVC	0.365 (0.369)	-	-	

References:

1. Seshasai et al. (2011). Diabetes mellitus, fasting glucose, and risk of cause-specific death. New England Journal of Medicine 364:829-841. doi: 10.1056/NEJMoa1008862
2. Moser et al. (2015). Modeling absolute differences in life expectancy with a censored skew-normal regression approach. PeerJ 3:e1162. doi: 10.7717/peerj.1162
3. Kim et al. (2017). Independent and joint associations of grip strength and adiposity with all-cause and cardiovascular disease mortality in 403,199 adults: The UK Biobank study. American Journal of Clinical Nutrition 106:773–782. doi: 10.3945/ajcn.117.156851
4. Alharbi et al. (2021). The association of weight change and all-cause mortality in older adults: a systematic review and meta-analysis. Age and Ageing 50: 697–704. doi: 10.1093/ageing/afaa231
5. Rostamian et al. (2022). Association of cognitive function with increased risk of cancer death and all-cause mortality: Longitudinal analysis, systematic review, and meta-analysis of prospective observational studies. PlosOne 7:e0261826. doi: 10.1371/journal.pone.0261826
6. Kim et al. (2023). Association of balance impairment with risk of incident cardiovascular diseases among older adults. European Journal of Medical Research 28:e455. doi: 10.1186/s40001-023-01426-7

7. Gil S. Araujo et al. (2024). Reduced Body Flexibility Is Associated With Poor Survival in Middle-Aged Men and Women: A Prospective Cohort Study. Scandinavian Journal of Medicine and Science in Sports 34: e14708. doi: 10.1111/sms.14708
8. Rydell et al. (2024). FEV1 and FVC as robust risk factors for cardiovascular disease and mortality: Insights from a large population study. Respiratory Medicine 27: e107614. doi: 10.1016/j.rmed.2024.107614

Supplementary Information: Note 4

A generalized approach for estimating the pace of aging

Marije H. Sluiskes, PhD[1,2], Joris Deelen, PhD[1], Hein Putter, PhD[1], Sara Hägg, PhD[2], and Mar Rodriguez-Girondo, PhD[*1]

[1]Biomedical Data Sciences, Leiden University Medical Center, Leiden, The Netherlands; [2]Medical Epidemiology and Biostatistics, Karolinska Institutet, Stockholm, Sweden

(m.rodriguez_girondo@lumc.nl)

This Note describes the definition of the health outcomes considered in Table 2 of the main manuscript (cardiovascular disease, diabetes and dementia) in more detail. This is done on a per-outcome basis.

Cardiovascular disease (CVD)

Information on time-to-CVD was obtained from the National Patient Register (including the Causes of Death Register and the Outpatient Register) linked to the Swedish Twin Register. When conducting the analyses that resulted in main manuscript Table 2, only prospective cases were considered (i.e. the Cox model was fitted on data from those still at risk for CVD at the latest assessment – see also Figure 1 of the main manuscript, where the red cross now represents diagnosis of CVD). This resulted in a

dataset of N = 549 individuals, of which 114 were diagnosed with CVD during follow-up. Follow-up was available until December 31, 2014.

Diabetes

Information on time-to-CVD was obtained from the National Patient Register (including the Causes of Death Register and the Outpatient Register) linked to the Swedish Twin Register.

When conducting the analyses that resulted in main manuscript Table 2, only prospective cases were considered (i.e. the Cox model was fitted on data from those still at risk for diabetes at the latest assessment – see also Figure 1 of the main manuscript, where the red cross now represents diagnosis of diabetes). This resulted in a dataset of N = 636 individuals, of which 49 were diagnoses with diabetes during follow-up. Follow-up was available until December 31, 2014.

Dementia

Information on time-to-dementia was obtained from all available sources for twins in the Swedish Twin Registry: the National Patient Register (including the Causes of Death Register and the Outpatient Register), the Prescribed Drug Register and clinical dementia diagnoses evaluated in the GENDER, OCTO-Twin, SATSA, HARMONY and SALZA studies (GOSH), estimated through patient and informant interviews and medical records.

For all 745 SATSA participants for whom the wPoA was calculated, both register and GOSH data was available. This likely leads to a more accurate estimation of age at onset (as the first symptoms generally start to show much earlier than registration of a dementia diagnosis in one of the registers occurs). Diagnoses included both vascular dementia and Alzheimer's disease. When conducting the analyses that resulted in main manuscript Table 2, only prospective cases were considered (i.e. the Cox model was fitted on data from those still at risk for diabetes at the latest assessment – see also Figure 1 of the main manuscript, where the red cross now represents diagnosis of diabetes). This resulted in a dataset of N = 681 individuals, of which 90 were diagnosed with dementia during follow-up. Follow-up was available until December 31, 2014.
